# Semaglutide for primary prevention of major adverse cardiac and cerebrovascular events in patients with type 2 diabetes and comorbid rheumatoid arthritis: a target-trial emulation

**DOI:** 10.1093/ehjcvp/pvag044

**Published:** 2026-06-23

**Authors:** Farheen Malik, Mandar Shah, Yu Chang, Ishmum Chowdhury, Sridhar Mangalesh, Ahmed Ashraf Morgan, Ezerioha Pascal, Vishakha Modak, Nachum Lebovics, Suraj Adhikari, Pei-Lun Lee, Wei-Cheng Chen, Kuan-Yu Chi

**Affiliations:** Department of Medicine, Jacobi Medical Center, Albert Einstein College of Medicine, Bronx, NY 10469, USA; Department of Medicine, Jacobi Medical Center, Albert Einstein College of Medicine, Bronx, NY 10469, USA; Section of Neurosurgery, Department of Surgery, National Cheng Kung University Hospital, Tainan 704302, Taiwan; Department of Medicine, Jacobi Medical Center, Albert Einstein College of Medicine, Bronx, NY 10469, USA; Department of Medicine, Jacobi Medical Center, Albert Einstein College of Medicine, Bronx, NY 10469, USA; Department of Medicine, Jacobi Medical Center, Albert Einstein College of Medicine, Bronx, NY 10469, USA; Department of Internal Medicine, Jacobi Medical Center/North Central Bronx, Albert Einstein College of Medicine, Bronx, NY 10467, USA; Department of Medicine, Jacobi Medical Center, Albert Einstein College of Medicine, Bronx, NY 10469, USA; Department of Medicine, Jacobi Medical Center, Albert Einstein College of Medicine, Bronx, NY 10469, USA; Department of Medicine, Jacobi Medical Center, Albert Einstein College of Medicine, Bronx, NY 10469, USA; Department of Medicine, Jacobi Medical Center, Albert Einstein College of Medicine, Bronx, NY 10469, USA; Department of Orthopedics, Shuang Ho Hospital, Taipei Medical University, New Taipei City 23561, Taiwan; Department of Medicine, Jacobi Medical Center, Albert Einstein College of Medicine, Bronx, NY 10469, USA

**Keywords:** Semaglutide, Rheumatoid arthritis, Diabetes, Primary prevention

## Abstract

**Aims:**

Rheumatoid arthritis (RA)-related physical limitations often hinder sustained physical activity and weight management, thereby amplifying cardiovascular risk through adverse metabolic profiles and chronic inflammation. We aimed to assess the effectiveness of semaglutide on the risk of incident major adverse cardiovascular and cerebrovascular events (MACCE) in obese adults with type 2 diabetes mellitus (T2DM) and RA in a primary-prevention setting.

**Methods and results:**

We emulated a target trial using data from the TriNetX US database, including obese adults [aged ≥18 years; body mass index (BMI) ≥30 kg/m^2^] with T2DM and comorbid RA and no prior history of stroke, heart failure (HF), acute coronary syndrome, or coronary revascularization. Using a new-user design, we compared patients initiating semaglutide with those initiating non-GLP-1 receptor agonist (GLP-1RA) second-line glucose-lowering therapies. Patients with contraindications to GLP-1RAs were excluded. Propensity-score matching (PS) (1:1) was used to balance baseline covariates. The primary outcome was incident MACCE, defined as a composite of all-cause mortality, myocardial infarction (MI), HF, or stroke. Secondary outcomes included individual MACCE components, HF hospitalization, and disease-modifying anti-rheumatic drug (DMARD) escalation, with Bonferroni correction applied for multiple comparisons. Hazard ratios (HRs) and 95% confidence intervals (CIs) were estimated using Cox proportional hazards models over a follow-up of up to 2 years. Between 1 January 2014 and 1 January 2025, we identified 1200 and 2972 patients who initiated semaglutide and non-GLP-1RA therapies, respectively, within 3 months of meeting eligibility. After PS matching, 1017 semaglutide users were compared with 1017 non-GLP-1RA users (mean age 59.5 vs. 59.3 years; women 81.3% vs. 81.0%; mean BMI 38.2 vs. 38.0 kg/m^2^; HbA1c, 6.8% vs. 7.0%). Semaglutide initiation was associated with a significantly lower risk of incident MACCE compared with non-GLP-1RA therapies (12.7% vs. 16.5%; HR, 0.75; 95% CI, 0.60–0.94; *P* = 0.01). This benefit was driven primarily by a lower risk of incident HF (8.9% vs. 12.5%; HR, 0.69, 95% CI, 0.53–0.91; *P* = 0·007). Semaglutide use was also associated with significantly lower DMARD escalation (16.6% vs. 21.6%; HR, 0.75; 95% CI, 0.60–0.90; *P* = 0.003). No significant differences were observed in all-cause mortality, MI, or stroke.

**Conclusion:**

Among obese adults with T2DM and comorbid RA, initiation of semaglutide was associated with a reduced risk of incident MACCE, driven predominantly by a reduction in incident HF, in a primary-prevention setting. Prospective studies are needed to confirm these observations and establish causality.

## Graphical Abstract

**Graphical Abstract:**
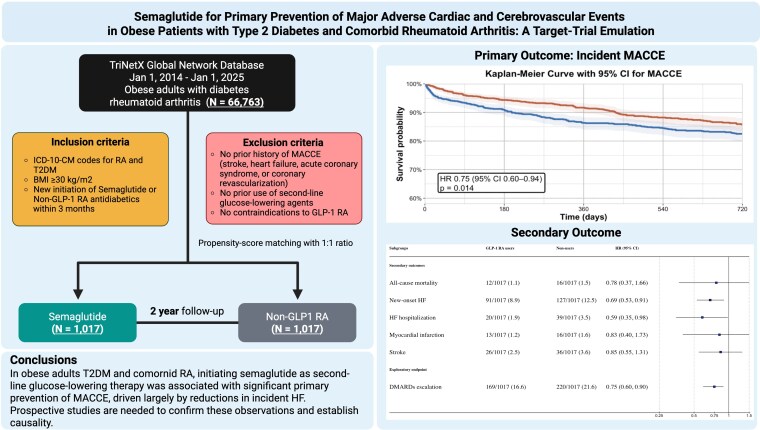

## Introduction

Individuals with rheumatoid arthritis (RA) face up to a two-times higher risk of developing atherosclerotic cardiovascular disease (ASCVD) events than the general population.^1^ This excess burden reflects the convergence of chronic systemic inflammation, an accelerator of atherosclerosis, alongside exposure to medications such as non-steroidal anti-inflammatory drugs (NSAIDs) and glucocorticoids, and a greater prevalence of traditional cardiometabolic risk factors.^2,3^ Obesity and RA increasingly coexist, and its prevalence with RA has been reported modestly higher than of the general population in some cohorts.^4,5^ Large population-based cohorts demonstrated that obesity [body mass index (BMI) ≥30 kg/m^2^] affects 21%–34% of individuals with RA, compared with 18%–25% in matched general-population comparators.^4,5^ Additionally, patients with RA may exhibit sarcopenic obesity, characterized by increased fat mass relative to lean mass despite similar BMI.^6^ In RA, obesity frequently clusters with hypertension, insulin resistance, and metabolic syndrome, compounding ASCVD risk.^3,7^ Additionally, RA-related physical limitations, driven by pain, stiffness, and functional impairment, often hinder sustained physical activity and weight management, thereby amplifying cardiovascular risk through adverse metabolic profiles and chronic inflammation.

Several observational studies suggest that obesity in RA is associated with higher disease activity and a lower likelihood of achieving remission, although an obesity paradox has also been described, whereby higher BMI was associated with lower radiographic progression and mortality in some cohorts.^8,9^ Nonetheless, active disease and increased biomechanical loading within joints may contribute to reduced physical activity and greater sedentary behaviour, which may in turn be associated with increased cardiovascular (CV) risk.^7,10^ Proposed mechanisms include both pharmacokinetic factors, such as altered drug distribution in individuals with greater adiposity, and biological amplification of inflammation mediated by adipose-derived cytokines and adipokines.^11^ Against this backdrop, glucagon-like peptide-1 receptor agonists (GLP-1RAs) are glucose-lowering agents with established weight-reducing, cardioprotective, and nephroprotective properties.^10^ Beyond these metabolic effects, GLP-1RAs demonstrate anti-inflammatory activity in humans, including suppression of cytokines such as IL-6, TNF, and IL-1β, suggesting a potential disease-modifying role in inflammatory arthropathies.^10^

Given these dual cardiometabolic and anti-inflammatory benefits, expanding the therapeutic role of GLP-1RAs in RA represents a timely and clinically meaningful opportunity. Yet despite compelling biological rationale and clinical need, their cardiovascular effectiveness in RA remains underexplored. To address this gap, we aimed to evaluate whether initiation of semaglutide as a second-line glucose-lowering therapy is associated with a reduced risk of major adverse cardiovascular events (MACCE) among obese adults with type 2 diabetes mellitus (T2DM) and comorbid RA.

## Methods

### Specification of the target trial

We emulated a pragmatic randomized trial employing a target-trial emulation (TTE) framework^7^ to examine the effect of semaglutide as a second-line glucose-lowering agent for the primary prevention of MACCE in obese patients with T2DM and comorbid RA with no prior history of stroke, heart failure (HF), acute coronary syndrome (ACS), or coronary revascularization. Aligned with the TTE framework, we pre-specified eligibility criteria, treatment strategies, assignment (Time 0), follow-up, and endpoints, with the intention-to-treat (ITT) as a causal contrast (see Supplementary material online, *Table S1*). This design aims to reduce confounding and immortal-time bias inherent to observational studies.^12^

### Eligibility criteria

Eligible participants are obese adult patients (≥18 years) with T2DM and RA (*Table 1*). Obesity is defined as a BMI ≥30 kg/m^2^. Participants with any prior use of common second-line glucose-lowering agents before index enrolment, including GLP-1RAs, dipeptidyl peptidase-4 (DPP-4) inhibitors, sodium-glucose co-transporter 2 inhibitors (SGLT2) inhibitors, sulfonylureas, thiazolidinediones (TZD), and insulin use, were excluded. To reflect real-world practice, metformin use was not required for eligibility, as some patients may have been managed with lifestyle modification alone, may not have tolerated metformin, or may have declined therapy. To align with the primary prevention strategy, we also excluded participants with a history of cardiomyopathy, HF, stroke, ACS, and coronary revascularization prior to baseline (Time 0).

**Table 1:** Baseline characteristics of patients with diabetes and RA before and after propensity-score matching

	Before propensity score matching			After propensity score matching			
Characteristic name	Semaglutide (*n* = 1200)	GLP-1RA (*n* = 2972)	*P*-value	Semaglutide (*n* = 1017)		GLP-1RA (*n* = 1017)	SMD
**Demographics**							
Age at Index (years)	58.3 ± 11.0	64.7 ± 11.5	<0.001	59.5 ± 10.8	59.3 ± 11.3	0.019	
Male, *n* (%)	198 (16.5)	990 (33.3)	<0.001	190 (18.7)	193 (19.0)	0.008	
Female, *n* (%)	1002 (83.5)	1979 (66.6)	<0.001	827 (81.3)	824 (81.0)	0.008	
Unknown gender, *n* (%)	70 (4.7)	126 (4.0)	0.22	51 (4.3)	54 (4.5)	0.01	
Asian	16 (1.3)	75 (2.5)	0.017	13 (1.3)	25 (2.5)	0.087	
Black or African American, *n* (%)	280 (23.3)	659 (22.2)	0.417	235 (23.1)	230 (22.6)	0.012	
White, *n* (%)	796 (66.3)	1882 (63.3)	0.067	666 (65.5)	657 (64.6)	0.019	
Other race, *n* (%)	39 (3.2)	111 (3.7)	0.446	37 (3.6)	37 (3.6)	0.000	
Unknown race, *n* (%)	52 (4.3)	210 (7.1)	<0.001	50 (4.9)	58 (5.7)	0.035	
Hispanic or Latino, *n* (%)	78 (6.5)	259 (8.7)	0.017	72 (7.1)	83 (8.2)	0.041	
Not Hispanic or Latino, *n* (%)	820 (68.3)	1936 (65.1)	0.049	684 (67.3)	665 (65.4)	0.040	
Unknown Ethnicity, *n* (%)	302 (25.2)	777 (26.1)	0.514	261 (25.7)	269 (26.4)	0.018	
Married, *n* (%)	332 (27.7)	813 (27.4)	0.838	274 (26.9)	277 (27.2)	0.007	
Divorced, *n* (%)	69 (5.8)	183 (6.2)	0.617	58 (5.7)	69 (6.8)	0.045	
BMI, mean (SD)	39.1 ± 7.3	34.9 ± 6.5	<0.001	38.2 ± 6.8	38.0 ± 7.4	0.030	
BMI ≥ 35 kg/m^2^, *n* (%)	858 (71.5)	1320 (44.4)	<0.001	678 (66.6)	672 (66.0)	<0.01	
**Diagnosis**							
Essential hypertension, *n* (%)	805 (67.1)	2214 (74.5)	<0.001	696 (68.4)	694 (68.2)	0.004	
Hyperlipidemia, *n* (%)	419 (34.9)	1237 (41.6)	<0.001	363 (35.7)	370 (36.4)	0.014	
Morbid obesity, *n* (%)	391 (32.6)	401 (13.5)	<0.001	270 (26.6)	269 (26.4)	0.002	
Sleep apnea, *n* (%)	350 (29.2)	497 (16.7)	<0.001	256 (25.2)	243 (23.9)	0.030	
Chronic kidney disease, *n* (%)	96 (8.0)	526 (17.7)	<0.001	91 (8.9)	91 (8.9)	0.000	
Atrial fibrillation and flutter, *n* (%)	53 (4.4)	291 (9.8)	<0.001	48 (4.7)	53 (5.2)	0.023	
Venous thromboembolism, *n* (%)	20 (1.7)	71 (2.4)	0.148	15 (1.5)	24 (2.4)	0.065	
Chronic lower respiratory diseases, *n* (%)	327 (27.2)	715 (24.1)	0.031	268 (26.4)	251 (24.7)	0.038	
Nicotine dependence	80 (6.7)	262 (8.8)	0.022	74 (7.3)	72 (7.1)	0.008	
Alcohol related disorders	10 (0.8)	37 (1.2)	0.254	10 (1.0)	10 (1.0)	0.000	
Systemic lupus erythematosus	48 (4.0)	84 (2.8)	0.050	37 (3.6)	32 (3.1)	0.027	
Sjögren syndrome	45 (3.8)	74 (2.5)	0.027	34 (3.3)	34 (3.3)	0.000	
Polymyalgia rheumatica	17 (1.4)	79 (2.7)	0.015	14 (1.4)	16 (1.6)	0.016	
**Medication**							
Statins, *n* (%)	448 (37.3)	1383 (46.5)	<0.001	389 (38.2)	399 (39.2)	0.020	
Ezetimibe, *n* (%)	28 (2.3)	88 (3.0)	0.264	26 (2.6)	31 (3.0)	0.030	
Fenofibrate, *n* (%)	21 (1.8)	77 (2.6)	0.105	19 (1.9)	24 (2.4)	0.034	
Metformin, *n* (%)	429 (35.8)	1244 (41.9)	<0.001	377 (37.1)	378 (37.2)	0.002	
Beta-blockers, *n* (%)	276 (23.0)	937 (31.5)	<0.001	247 (24.3)	223 (21.9)	0.056	
Angiotensin-II inhibitors, *n* (%)	266 (22.2)	753 (25.3)	0.031	238 (23.4)	243 (23.9)	0.012	
ACE inhibitors, *n* (%)	179 (14.9)	688 (23.1)	<0.001	167 (16.4)	152 (14.9)	0.041	
Calcium channel blockers, *n* (%)	231 (19.2)	717 (24.1)	<0.001	206 (20.3)	202 (19.9)	0.010	
Potassium-sparing diuretics, *n* (%)	68 (5.7)	196 (6.6)	0.265	54 (5.3)	59 (5.8)	0.021	
Anticoagulants, *n* (%)	137 (11.4)	501 (16.9)	<0.001	113 (11.1)	117 (11.5)	0.012	
Aspirin, *n* (%)	91 (7.6)	405 (13.6)	<0.001	85 (8.4)	80 (7.9)	0.018	
Prednisone, *n* (%)	322 (26.8)	734 (24.7)	0.151	259 (25.5)	265 (26.1)	0.013	
Methylprednisolone, *n* (%)	292 (24.3)	508 (17.1)	<0.001	220 (21.6)	232 (22.8)	0.028	
Methotrexate, *n* (%)	201 (16.8)	477 (16.1)	0.579	174 (17.1)	168 (16.5)	0.016	
Hydroxychloroquine, *n* (%)	178 (14.8)	329 (11.1)	<0.001	141 (13.9)	127 (12.5)	0.041	
Leflunomide, *n* (%)	36 (3.0)	98 (3.3)	0.622	30 (3.0)	34 (3.3)	0.023	
TNF-alpha inhibitors, *n* (%)	154 (12.8)	210 (7.1)	<0.001	118 (11.6)	86 (8.5)	0.105	
Abatacept, *n* (%)	28 (2.3)	31 (1.0)	0.001	21 (2.1)	14 (1.4)	0.053	
**Laboratory**							
Hemoglobin A1c, %, mean ± SD	6.7 ± 1.2	7.4 ± 1.5	<0.001	6.8 ± 1.3	7.0 ± 1.4	0.08	
≥10.0%, *n* (%)	50 (3.4)	187 (5.9)	<0.001	48 (4.0)	46 (3.9)	<0.01	
ESR, mean ± SD, mm/h	27.4 ± 22.0	29.1 ± 23.9	0.205	27.6 ± 22.7	28.6 ± 22.7	0.045	
≥100 mm/h, *n* (%)	13 (0.9%)	36 (1.1%)	0.013	11 (0.9%)	10 (0.8%)	0.827	
GFR, mL/min/1.73m^2^, mean ± SD	80.4 ± 22.8	74.8 ± 26.8	<0.001	79.3 ± 22.6	80.6 ± 27.1	0.054	
GFR <60 mL/min/1.73m^2^, *n* (%)	242 (20.1%)	975 (32.8%)	<0.001	220 (21.6%)	225 (22.1%)	0.011	
LDL cholesterol, mg/dL, mean ± SD	95.7 ± 37.5	87.6 ± 35.2	<0.001	92.2 ± 34.5	93.9 ± 36.6	0.049	
≥100 mg/dL	285 (24.5)	8117 (15.2)	<0.001	306 (25.7)	316 (26.5)	0.01	
**Visit**							
Inpatient encounter, *n* (%)	78 (6.5)	280 (9.4)	0.002	67 (6.6)	62 (6.1)	0.020	
Emergency encounter, *n* (%)	252 (21.0)	623 (21.0)	0.978	207 (20.4)	205 (20.2)	0.005	

The relatively low proportion of metformin use (37.1% vs. 37.2%) likely reflects real-world variation in first-line diabetes management, including patients who were managed with lifestyle modification alone, those who declined metformin, and those for whom alternative treatment strategies were selected by the treating clinician. However, these information is not available within TriNetX.

ACE, angiotensin-converting enzyme; BMI, body mass index; DPP-4, dipeptidyl peptidase-4; GLP-1RA, glucagon-like peptide-1 receptor agonist; LDL, low-density lipoprotein; RA, rheumatoid arthritis; SGLT2, sodium-glucose co-transporter 2; SMD, standardized mean difference.

### Treatment strategies and assignment (target trial)

After meeting eligibility criteria, participants in the target trial are randomly assigned at baseline (Time 0) in a 1:1 ratio to one of two glucose-lowering treatment strategies: (i) intervention group: semaglutide; (ii) comparator group: non-GLP-1RA therapy, including DPP4 inhibitors, SGLT2 inhibitors, sulfonylurea, and TZD. We restricted the intervention to semaglutide to minimize within-class treatment heterogeneity and because semaglutide had the greatest contemporary uptake during the study period,^13^ likely owing to its greater glycemic and weight-loss efficacy. Tirzepatide was not included because its more recent introduction resulted in limited uptake and insufficient longitudinal follow-up during the study period. Eligible participants and their clinicians were aware of their assignments, consistent with an open-label design typical for pragmatic trials emulated with observational data. In accordance with an ITT effect as the causal contrast, treatment strategies were assigned at baseline, irrespective of subsequent medication adherence, cross-over, or add-on therapies.

### Study endpoints and follow-up (target trial)

The primary endpoint is MACCE, defined as the composite of all-cause mortality, new-onset HF, incident myocardial infarction (MI), or any stroke. To ensure that HF events represented new-onset diagnoses, we excluded patients with a history of HF before the index date, including those with documented left ventricular ejection fraction <50%. Secondary endpoints are individual components of the primary outcome and HF hospitalization, defined as the use of intravenous diuretics. We also exploratorily looked the rate of into disease-modifying anti-rheumatic drug (DMARD) escalation, defined as the use of biological DMARDs, including tumour necrosis factor (TNF)-alpha inhibitors, IL-6 receptor antagonists, abatacept, and rituximab. The study index date (Time 0) is the date of treatment randomization. Follow-up commenced at Time 0 and continued until the occurrence of the primary endpoint, loss of follow-up, or up to 2 years from Time 0, whichever occurred first.

### Statistical analysis

The primary analysis in the target trial uses Kaplan–Meier methods to estimate survival curves for the primary outcome up to 2 years from Time 0. Cox proportional hazards regression models would be used to estimate hazard ratios (HRs) with 95% confidence intervals (CIs), comparing participants assigned to semaglutide vs. the no GLP-1RA strategy.

## Emulation of the target trial

### Data source

We utilized the TriNetX US Collaborative Network, a federated network of 70 healthcare organizations (HCOs) aggregating de-identified, real-time electronic health records (EHR), to emulate the pre-specified target trial. TriNetX’s data were validated for data completeness, and there have been numerous studies successfully conducted on comparative effectiveness research using TriNetX,^14–17^ including in the context of cardiovascular disease research.^13,18,19^ The details regarding TriNetX are presented in Supplementary material online, *Methods S1*.

The TriNetX database operates under the Health Insurance Portability and Accountability Act (HIPAA) Privacy Rule as a limited data set, which means that all patient data in the network is de-identified in compliance with HIPAA standards.^12^ Thus, they exclude direct patient identifiers such as names, social security numbers, and contact information, ensuring the protection of patient privacy. This research was approved by the Institutional Review Board of National Cheng Kung University Hospital (NCKU-IRB Approval No. A-ER-114-687).

### Study population and definition of Time 0

We applied the pre-specified eligibility criteria within the TTE framework (see Supplementary material online, *Figure S1*) to identify obese patients, defined as a BMI ≥30 kg/m^2^, who received diagnostic codes of T2DM and RA between 1 January 2014, and 1 January 2025 (*Table 1*). To minimize misclassification of RA diagnosis, patients were required to have two separate RA diagnostic codes at least 6-month apart. Additionally, BMI was defined using the variable (LOINC 39156-5), which includes directly recorded BMI values. When BMI was not directly available, it was calculated from recorded height and weight measurements using the formula weight (kg)/height^2^ (m^2^). For adults, height from the same date as weight was used when available; otherwise, the last known adult height was applied.

Eligible patients were then subdivided into the intervention group and the comparator group. Employing a new-user approach, the intervention group consisted of patients who initiated their first-ever semaglutide, without sulfonylureas, DPP4 inhibitors, SGLT2 inhibitors, TZD, or insulin in the database within 3 months of meeting eligibility criteria. The comparator group consisted of patients who initiated non-GLP-1RA glucose-lowering agents over the same 3-month period. The 3-month window for treatment initiation was used to emulate a treatment assignment period while minimizing immortal time bias. Time 0 (index date) was defined as the date of treatment initiation: the first semaglutide initiation for the intervention group and non-GLP-1RA glucose-lowering therapies for the comparator group. To preserve new-user design and primary prevention setting, patients with records of second-line glucose-lowering therapies and MACCE in the database were excluded.

Follow-up for commenced at each patient’s Time 0 and continued until the occurrence of an outcome of interest, death, loss to follow-up (e.g. end of data availability for the patient in the TriNetX network), or up to 2 years from Time 0, whichever occurred first. The ICD-10-CM codes used to define the study cohort (see Supplementary material online, *Methods S1*) and endpoints (see Supplementary material online, *Methods S2*) are presented in the Supplement.

### Statistical analysis

To emulate randomization and control for baseline confounding, we applied 1:1 propensity-score (PS) matching using a greedy nearest-neighbour algorithm with a calliper width of 0.1 pooled SDs of the logit of the PS. The PS, representing the estimated probability of initiating semaglutide vs. non-GLP-1RA glucose-lowering agents, given baseline characteristics, was estimated using a logistic regression model. Covariates included in the PS model were selected *a priori* based on clinical relevance, prior literature, and their plausible roles as baseline confounders of the association between GLP-1RA initiation and cardiovascular outcomes: age, sex, race, ethnicity, hypertension, hyperlipidaemia, smoking, chronic obstructive pulmonary disease, chronic kidney disease (CKD), sleep apnoea, morbid obesity, Sjögren syndrome, metformin, methotrexate, hydroxychloroquine, TNF-alpha inhibitors, methylprednisolone, prednisone, aspirin, statins, ezetimibe, beta-blockers, angiotensin-converting enzyme inhibitors (ACEi), angiotensin receptor blockers (ARB), calcium channel blockers, anticoagulants, haemoglobin A1c, serum LDL, BMI, eGFR, serum erythrocyte sedimentation rate (ESR), and inpatient visit. Baseline covariates used for PS matching were assessed during the 12-month look-back period preceding the index date (Time 0). Baseline characteristics between the two groups were compared using standardized mean differences (SMDs) after PS matching, with an SMD <0.10 considered to indicate a negligible imbalance. Descriptive statistics for baseline characteristics were reported for continuous variables as mean (standard deviation) and for categorical variables as count (percentage). Because individual-level data cannot be exported from the platform, formal tests of normality were not performed. Pre-matching, continuous variables were compared using independent-sample *t*-tests and categorical variables using chi-square tests. Post-matching, SMDs were the primary metric for assessing balance.

Time-to-event analyses were performed using Cox proportional hazards models to estimate HRs and 95% CI for the primary and secondary outcomes. The proportional hazards assumption was assessed using the scaled Schoenfeld residual test described by Grambsch and Therneau *et al.*^20^ a default method implemented in the TriNetX analytics platform. This test evaluates whether the estimated regression coefficient varies over time. For the pre-specified secondary endpoints, a Bonferroni correction was applied to account for multiple hypothesis testing across five outcomes (all-cause mortality, MI, stroke, HF, and HF hospitalization), with a corrected *P*-value threshold of <0.01 (0.05/5) indicating statistical significance.

We also conducted a per-protocol (PP) analysis by restricting the cohort to individuals who remained adherent to their initial treatment assignment at a 1-year landmark. In this analysis, GLP-1RA users were required to have at least one additional prescription between 6 and 12 months following the index date to account for potential underfilling or early discontinuation; the comparator group continued to remain free of GLP-1RA records at a 1-year landmark. To further investigate whether RA status modified the effect of GLP-1RA, we constructed an additional cohort of patients with T2DM without comorbid RA (see Supplementary material online, *Methods S4*) and compared the association of GLP-1RA use with outcomes across the RA and non-RA cohorts.

To explore the potential impact of residual confounding on our findings, we utilized two exploratory approaches. First, we set urinary tract infection (UTI) and fracture as falsification endpoints. Second, *E*-values were computed for the study endpoints’ point estimate and the lower bound of its 95% CI to quantify the minimum strength of association an unmeasured confounder would need to have with both intervention initiation and the outcome (conditional on measured covariates) to fully explain away the observed effect. All analyses were conducted using the TriNetX Advanced Analytics platform. Two-sided *P*-values <0.05 were considered statistically significant for the primary outcome and falsification endpoints, unless otherwise adjusted for multiple comparisons.

## Results

**Figure 1:**
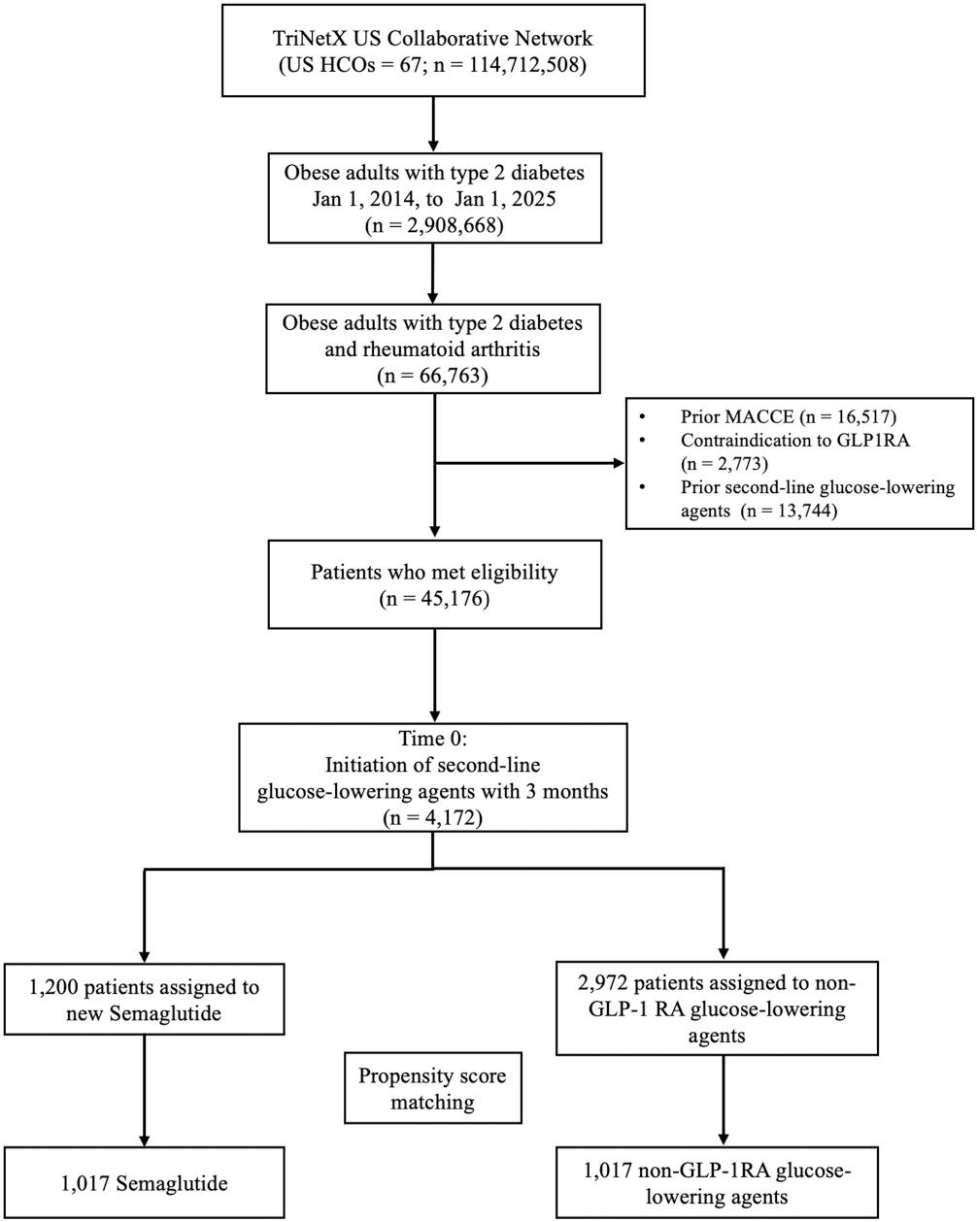
Patient recruitment flowchart depicting inclusion and exclusion criteria.

Between 1 January 2014 and 1 January 2025, we identified 2 908 668 obese adults with T2DM. Among which, 66 763 had comorbid RA. The emulated eligibility criteria further identified 45 176 patients and 5531 of which initiated second-line glucose-lowering agents within 3 months of meeting eligibility (semaglutide = 1200; non-GLP-1RA = 2972) (*Figure 1*). After PS matching, 1017 semaglutide users were matched to 1017 non-GLP-1RA therapy, with balanced covariates between two groups (mean age 59.5 vs. 59.3 years; women 81.3% vs. 81.0%; mean BMI 38.2 vs. 38.0 kg/m^2^; HbA1c, 6.8% vs. 7.0%). (all SMDs <0.10) (*Table 1*). A total of 80 patients (7.8%) in the semaglutide group also received SGLT2 inhibitors.

### Primary endpoint

**Figure 2:**
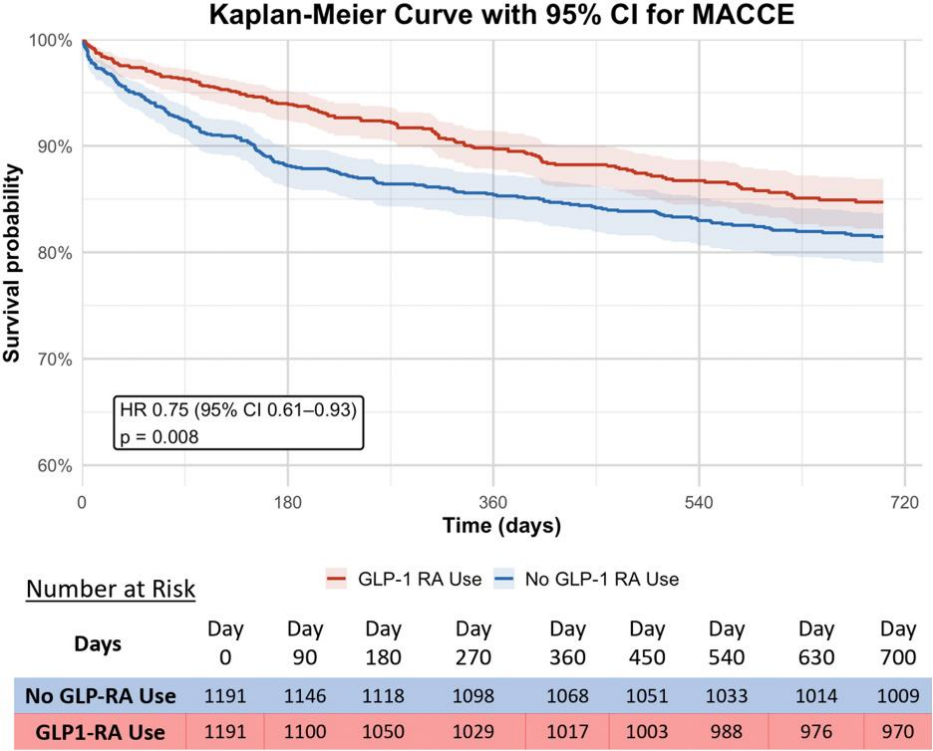
Kaplan–Meier survival curves for comparative risk of major adverse cardiovascular events between GLP-1 receptor agonist use and no use in obese patients with diabetes and rheumatoid arthritis.

Over the study follow-up period (median days: intervention, 730; control, 730), in the matched cohort, the composite of MACCE occurred in 129 (12.7%) patients in the intervention vs. 168 (16.5%) in the control group (HR, 0.75; 95% CI, 0.60–0.94; *P* = 0.01; *E*-value: 2.00) (*Figure 2*).

### Secondary endpoints

Semaglutide use was associated with significant lower risk of incident HF [8.9% vs. 12.5%; HR, 0.69; 95% CI, 0.53–0.91; *P* = 0.007 (<0.01 after Bonferroni correction); *E*-value: 2.26] and DMARDs escalation [16.6% vs. 21.6%; HR, 0.75; 95% CI, 0.60–0.90; *P* = 0.003 (<0.01 after Bonferroni correction); *E*-value: 2.00]. There was no significant difference in HF hospitalization [1.9% vs. 3.5%; HR, 0.59; 95% CI, 0.35–0.98; *P* = 0.04 (>0.01 after Bonferroni correction)], all-cause mortality (1.1% vs. 1.5; HR, 0.78; 95% CI, 0.37–1.66; *P* = 0.18), MI (1.2% vs. 1.6%; HR, 0.83; 95% CI, 0.40–1.73; *P* = 0.62), and any stroke (2.5% vs. 3.6%; HR, 0.85; 95% CI, 0.55 to 1.31; *P* = 0.47) (*Table 2*).

**Table 2:** Cox proportional hazard analysis for Semaglutide use and study endpoints in the matched cohort

	Semaglutide (*n* = 1017)	Non-GLP-1RA glucose-lowering agents (*n* = 1017)	HR (95% CI)	*P*-value (log-rank)	*E*-value (LNL)
*Primary endpoint*					
MACCE, *n* (%)	129 (12.7)	168 (16.5)	0.75 (0.60–0.94)	0.01	2.00 (2.72)
*Secondary endpoints*					
All-cause mortality, *n* (%)	12 (1.1)	16 (1.5)	0.78 (0.37–1.66)	0.53	1.88 (4.85)
Incident HF, *n* (%)	91(8.9)	127 (12.5)	0.69 (0.53–0.91)	0.008*	2.26 (3.18)
HF hospitalization, *n* (%)	20 (1.9)	39 (3.5)	0.59 (0.35–0.98)	0.04	
Myocardial infarction, *n* (%)	13 (1.2)	16 (1.6)	0.83 (0.40–1.73)	0.62	
Any stroke, *n* (%)	26 (2.5)	36 (3.6)	0.85 (0.55–1.31)	0.47	
*Exploratory endpoints*					
DMARDs escalation	169 (16.6)	220 (21.6)	0.75 (0.60–0.90)	0.003	
*Falsification endpoints*					
Urinary tract infection, *n* (%)	85 (8.4)	93 (9.1)	0.93 (0.69–1.25)	0.65	
Fracture, *n* (%)	54 (5.3)	55 (5.4)	1.00 (0.69–1.47)	0.96	

CI, confidence interval; DMARD, disease-modifying anti-rheumatic drugs; GLP-1RA, glucagon-like peptide-1 receptor agonist; HF, heart failure; HR, hazard ratio; LNL, lower normal limit; MACCE, major adverse cardiovascular and cerebrovascular events.

^*^ *P* < 0.01 after Bonferroni correction.

### Falsification endpoints

There were no differences in UTI (HR, 0.93; 95% CI, 0.69–1.25; *P* = 0.65) and any fracture (HR, 1.00; 95% CI, 0.69–1.47; *P* = 0.96) (*Table 2*).

### Per-protocol analyses

At a 1-year landmark, 794 patients remained on GLP-1RA, and 2577 patients remained free of GLP-1RA. After PS matching, those who adhered to GLP-1RA experienced a significant reduction in all-cause death or HF hospitalization (HR, 0.70; 95% CI, 0.57–0.86; *P* = 0.001), which also appeared to be driven by HF hospitalization (HR, 0.68; 95% CI, 0.55–0.85; *P* = 0.001). There was no significant difference in other endpoints (see Supplementary material online, *Table S2*).

### Rheumatoid arthritis vs. non-rheumatoid arthritis

Applying the same eligibility criteria to construct a non-RA cohort, we identified a total of 127 106 obese adults with T2DM without RA who initiated semaglutide as second-line glucose-lowering agents. After 1:1 PS matching, in patients who received semaglutide, RA patients experienced a significantly lower rates of incident MACCE (12.0% vs. 16.9%; HR, 0.68; 95% CI, 0.51–0.91; *P* = 0.008) (see Supplementary material online, *Figure S2*), also primarily driven by reductions in new-onset HF (9.3% vs. 12.6%; HR, 0.70; 95% CI, 0.55–0.89; *P* = 0.003) (see Supplementary material online, *Table S3*).

## Discussion

Our study found that semaglutide initiation was associated with a significant 25% reduction in new-onset MACCE, driven primarily by a substantially lower risk of incident HF, when compared with non-GLP-1RA second-line glucose-lowering therapies among obese patients with T2DM and comorbid RA. However, no difference was observed in all-cause mortality, MI, or stroke events. It is possible that our study population reflected a relatively lower-risk, primary prevention setting, in which absolute rates of atherosclerotic events were low, thereby limiting statistical power to detect differences in these individual outcomes, an outcome pattern consistent with a prior primary prevention analysis for GLP-1RA.^18^ In addition, the relatively short 2-year follow-up may have further limited the number of accrued events, limiting statistical power. This was further reflected by the outcome of HF hospitalization that did not reach statistical significance after Bonferroni correction, likely owing to low absolute event rate (1.9% vs. 3.5%). Comparable falsification endpoints between two treatment groups alongside calculated *E*-values renders study endpoints less vulnerable to unmeasured confounding accounted for the observed associations. Interestingly, while most evidence supporting the cardiovascular benefit of GLP-1RA is mainly derived from secondary-prevention trials in patients with established ASCVD,^21^ our findings suggest that GLP-1RA may confer important primary-prevention advantages in this high-risk inflammatory population. Given that patients with RA experience a 50%–70% increased risk of CV mortality,^22^ the observed cardioprotective benefits of semaglutide in our study are particularly relevant.

Our cohort had a mean BMI exceeding 38, with over 60% of participants having a BMI ≥35, consistent with morbid obesity. This degree of adiposity is clinically meaningful as obesity in RA is consistently associated with higher disease activity, reduced functional status, lower likelihood of achieving remission, and higher CV morbidity.^10,23^ These complex interplays render weight-loss intervention an important therapeutic target in RA. Notably, bariatric surgery studies have demonstrated consistent improvements in RA disease activity, higher remission rates, reduced disease-modifying anti-rheumatic drug and corticosteroid requirements, and sustained reductions in inflammatory markers.^24,25^ However, surgical interventions are not always feasible for RA patients given their unique perioperative challenges arising from several factors such as chronic immunosuppressive therapy and increased infection risk.^26^ In this context, our finding that DMARD escalation was significantly with semaglutide use is notable, as it raises the possibility that GLP-1RA may favourably influence RA disease control, although this observation should be interpreted cautiously given the limitations of EHR-based treatment-intensity measures. This also echoes a recent study suggesting GLP-1RA use was associated with improvements in disease activity and reductions in serum levels of C-reactive protein and erythrocyte sedimentation rate, and patient-reported symptoms, partially independent of weight loss.^27^

Furthermore, Wang *et al.* recently showed a significant 24% lower risk of thromboembolic events and reduced all-cause mortality among GLP-1RA users compared with those initiating DPP-4 inhibitors.^28^ Similarly, Karacabeyli *et al.* reported substantial reductions in all-cause mortality and MACCE among individuals with immune-mediated inflammatory diseases initiating GLP-1RAs.^29^ Building upon these observations, our study extends this literature by specifically evaluating obese patients with T2DM and comorbid RA. In an additional analysis including a matched non-RA T2DM cohort receiving semaglutide, patients with RA had significantly lower rates of MACCE and incident HF than those without RA. Although this exploratory comparison should not be interpreted as definitive proof of effect modification by RA status, it suggests that the cardiovascular benefit of semaglutide is preserved in, and may be particularly relevant to, this inflammatory high-risk population. Collectively, these studies reinforce the potential broader cardiovascular and systemic benefits of GLP-1RA across inflammatory conditions and support the biological plausibility of the associations observed in our RA cohort.

Prior randomized trials and observational studies demonstrate that the cardiovascular benefits of GLP-1RA are most pronounced among individuals with obesity. In the SUSTAIN-6 trial, semaglutide reduced 3-point MACCE among patients with T2DM at high cardiovascular risk, with benefit driven primarily by reductions in nonfatal stroke, while the SELECT trial extended these findings to overweight or obese patients with established cardiovascular disease without diabetes. In a large real-world cohort, Chen *et al.* similarly reported lower rates of MACCE with GLP-1 RA compared with DPP-4 inhibitors, with benefit confined to patients with BMI ≥25 and largely driven by reductions in cardiovascular death and HF hospitalization. Consistent with these observations,^30–32^ our findings extend this pattern of obesity-modified cardiovascular benefit to patients with RA, with a signal driven predominantly by HF outcomes. Looking forward, newer incretin-based therapies may further expand this treatment landscape. SURMOUNT-5 showed greater weight-loss efficacy with tirzepatide than with semaglutide in obesity,^33^ highlighting the need for future studies evaluating whether tirzepatide confer incremental cardiometabolic benefit in patients with T2DM and comorbid RA.

Evidence for HF benefit has been particularly robust. RA is associated with a markedly higher HF burden, often two- to four-fold greater than the general population, and recent HF-focused trials, including STEP-HFpEF, STEP-HFpEF DM, and SUMMIT, have shown that GLP-1RA therapy significantly reduces HF events and cardiovascular death across diverse HF phenotypes, irrespective of diabetes status.^34–37^ These findings closely mirror our observed 30% reduction in incident HF and 40% reduction in HF hospitalizations, representing among the strongest signals in our study. The preferential HF benefit may be explained by improvements in cardiac structure, congestion, and functional capacity, as well as emerging mechanistic insights supporting the adipokine hypothesis, which posits that excess and inflamed adipose tissue promotes HF, particularly HFpEF, through dysregulated adipokine signalling, systemic inflammation, myocardial fibrosis, and impaired diastolic relaxation. GLP-1RA therapy favourably modulates adipokine profiles and reduces adipose-driven inflammatory burden, mechanisms that may be especially relevant in our cohort with a mean BMI of 37.^38^

### Limitations

Our study has several limitations. First, its observational design inherently limits causal inference. Second, the absence of individual-level patient data precluded detailed assessments of medication dosage, titration, adherence, or treatment crossover. Additionally, duration of diabetes was not available within the database and therefore could not be incorporated into the PS model. Although restricting the cohort to patients initiating second-line glucose-lowering therapy and balancing baseline HbA1c levels likely reduced heterogeneity in diabetes severity, residual confounding related to differences in diabetes duration cannot be completely excluded. Furthermore, because individual-level data were not accessible, more flexible survival modelling and direct visualization of hazard functions were not feasible. Third, the comparator group included SGLT2 inhibitors, which have established cardiovascular benefits. As such, the comparator represented an active rather than neutral treatment strategy, which may have attenuated observable differences in harder cardiovascular outcomes, particularly MI and stroke, and biased the treatment contrast towards the null. Fourth, the inclusion of obese patients with the elevated BMI in this cohort may limit the generalizability of our findings to patients with RA without obesity. Fifth, constraints of the TriNetX Analytics Platform prevented 1:2 or 1:3 propensity score matching, yielding a relatively small, matched sample that precluded meaningful subgroup analyses. As PS matching retains only patients with adequate covariate overlap, the final analytic cohort represented a selected subset of the eligible population. This approach improved baseline comparability but excluded a substantial proportion of otherwise eligible patients, limiting generalizability. While alternative approaches, such as inverse probability weighting and other g-methods,^39,40^ could preserve more eligible patients, these are not implemented within the TriNetX Platform. Sixth, the Fine–Gray model is not available to account for competing risk of death. While inclusion of death within the composite MACCE endpoint may partially reduce this concern for MACCE, the possibility of competing-risk bias for individual non-fatal secondary outcomes remains. Seventh, reliance on ICD-10-CM codes to define the cohort and clinical endpoints introduces the possibility of diagnostic misclassification. Although we strengthened the RA definition by requiring 2 diagnosis codes at least 6 months apart, residual misclassification remains possible. T2DM was also defined using diagnostic codes, and HbA1c was not required for inclusion. Although all included patients subsequently initiated glucose-lowering therapy at the index date, residual misclassification of diabetes status cannot be excluded. Finally, because follow-up in TriNetX depends on continued capture of patient data within participating health care organizations and linked sources, informative censoring cannot be excluded. Patients who transfer care, lose insurance coverage, or otherwise leave the contributing system may have incomplete follow-up, and such information censoring may be associated with underlying cardiovascular risk.

## Conclusion

Initiating semaglutide as second-line glucose-lowering therapy was associated with significant primary prevention of MACCE, driven largely by reductions in incident HF, in obese adults with T2DM and comorbid RA. Future prospective randomized clinical trials are needed to validate these observations and to elucidate the immunometabolic mechanisms that may underlie the cardioprotective effects of GLP-1 RAs in inflammatory arthritis.

## Supplementary Material

pvag044_Supplementary_Data

## Data Availability

The data supporting the study findings are available from the TriNetX Analytics Network (www.trinetx.com) which includes subscription only access to de-identified data.
