# Supplementary material for: Semaglutide for primary prevention of major adverse cardiac and cerebrovascular events in patients with type 2 diabetes and comorbid rheumatoid arthritis: a target-trial emulation
Source: Eur Heart J Cardiovasc Pharmacother. 2026 Jun 23;12(5):368–78. doi: 10.1093/ehjcvp/pvag044 (PMC13588637; doi:10.1093/ehjcvp/pvag044)
Supplement: Supplementary file 1 [file pvag044_supplementary_data.docx]

**Supplementary file**

The following material supplements the article:

*”* *Semaglutide for Primary Prevention of Major Adverse Cardiac and Cerebrovascular Events in Obese Patients with Type 2 Diabetes and Comorbid Rheumatoid Arthritis: A Target-Trial Emulation”*

**eMethods 1.** Data Source

The participating HCOs within TriNetX are based in the United States, covering diverse geographic regions, including 39% from the South, 22% from the Northeast, 16% from the Midwest, 13% from the West, and 10% from unspecified areas. TriNetX standardizes data using common terminologies such as International Classification of Diseases, 10th Revision, Clinical Modification (ICD-10-CM), Current Procedural Terminology (CPT), RxNorm, and Logical Observation Identifiers Names and Codes (LOINC), and allows scalable cohort construction and statistical analysis. Race and ethnicity are self-reported at clinical encounters; missing demographic fields are classified as “unknown.” BMI was defined using the TriNetX BMI variable (LOINC 39156-5), which includes both directly recorded BMI values and TriNetX-derived BMI values. When BMI was not directly available, TriNetX calculated BMI from recorded height and weight measurements using the formula weight (kg)/height² (m²). For adults, height from the same date as weight was used when available; otherwise, the last known adult height was applied. For mortality data, the term "Deceased" was used to capture death. Mortality data in TriNetX is primarily derived from HCO death records, which are sourced from EHRs. TriNetX additionally supplements this by connecting to other sources, including billable codes from closed claims, the Social Security Administration Master Death File, private obituaries, and private claims. Mortality data directly from HCOs uses the ICD-10-CM code R99 to map the "Deceased" term. For patients with additional mortality data from the aforementioned external sources, these are also mapped to the "Deceased" term to improve data completeness. Researchers build cohort queries on the TriNetX online portal using these standardized terminologies. Once a cohort is designed, the query is processed by TriNetX's Advanced Analytics Platform, which provides built-in statistical tools for analysis.

Regarding missing data, TriNetX does not routinely perform comprehensive imputation of missing clinical values; beyond limited system-level derivations, missing values remain unaltered from the source electronic health records. Therefore,

TriNetX does not impute or estimate missing clinical values, except in four specific cases:

- Patient Encounters

If an observation is recorded with an encounter ID that does not match any ID on the HCO’s encounter list, TriNetX derives an encounter to accommodate these observations. If multiple observations share the same unmatched encounter ID, a single derived encounter is created, with the earliest observation date assigned as the start date and the latest as the end date. However, the encounter type is not inferred.

- Missing Start or End Dates for Encounters

If an HCO-provided encounter is missing a start date, TriNetX assigns the earliest start date from all associated observations. If an end date is missing, the latest date from related observations is assigned.

- Glomerular Filtration Rate (GFR) Estimation

Estimated GFR values are derived when serum, plasma, or blood creatinine levels are recorded, provided the patient’s sex and age are known. For pediatric patients, a height measurement within one year of creatinine testing is required.

- Oncology Diagnoses

TriNetX maps oncology diagnoses from ICD-O codes to ICD-10-CM using the National Cancer Institute’s Surveillance, Epidemiology, and End Results (SEER) program guidelines. Diagnoses derived through this mapping are flagged accordingly. Beyond these exceptions, missing data remain unaltered in the dataset, reflecting the original records as documented by the HCOs.

**eMethods 2. Cohort Query**

### Query Criteria for Cohort 1 (query name: Semaglutide)

This query was run on the network US Collaborative Network with 67 HCO(s) queried and 67 HCO(s) responded. A total of 45 provider(s) responded with patients. The final cohort included 1,200 patients who matched the query criteria listed in the table below.

| Group 1 | | | | | |
| --- | --- | --- | --- | --- | --- |
|  | **Group 1A** | | | | |
|  | must have | any of | diagnosis | UMLS:ICD10CM:M06 | Other rheumatoid arthritis |
|  |  |  | diagnosis | UMLS:ICD10CM:M05 | Rheumatoid arthritis with rheumatoid factor |
|  |  | and | laboratory | TNX:9083 | BMI (at least 30.00 kg/m2) |
|  |  | and | diagnosis | UMLS:ICD10CM:E11 | Type 2 diabetes mellitus |
|  | date constraint | | The terms in this group occurred between Jan 1, 2014 and Jan 1, 2025 | | |
|  | event relationship | | Any instance of Group 1B occurred within 3 months on or after any instance of Group 1A | | |
|  | **Group 1B** | | | | |
|  | must have |  | medication | NLM:RXNORM:1991302 | semaglutide |
|  | cannot have |  | medication | NLM:ATC:A10BH | Dipeptidyl peptidase 4 (DPP-4) inhibitors |
|  |  | or | medication | NLM:RXNORM:2601723 | tirzepatide |
|  |  | or | medication | NLM:RXNORM:1440051 | lixisenatide |
|  |  | or | medication | NLM:RXNORM:1534763 | albiglutide |
|  |  | or | medication | NLM:RXNORM:1551291 | dulaglutide |
|  |  | or | medication | NLM:RXNORM:60548 | exenatide |
|  |  | or | medication | NLM:RXNORM:475968 | liraglutide |
|  |  | or | visit | UMLS:HL7V3.0:VisitType:IMP | Visit: Inpatient Encounter |
|  |  | or | medication | NLM:VA:HS501 | INSULIN |
|  |  | or | medication | NLM:ATC:A10BK | Sodium-glucose co-transporter 2 (SGLT2) inhibitors |
|  |  | or | medication | NLM:ATC:A10BB | Sulfonylureas |
|  |  | or | medication | NLM:ATC:A10BG | Thiazolidinediones |
| Group 2 | | | | | |
|  | **Group 2A** | | | | |
|  | must have | any of | diagnosis | UMLS:ICD10CM:M06 | Other rheumatoid arthritis |
|  |  |  | diagnosis | UMLS:ICD10CM:M05 | Rheumatoid arthritis with rheumatoid factor |
|  |  | and | laboratory | TNX:9083 | BMI (at least 30.00 kg/m2) |
|  |  | and | diagnosis | UMLS:ICD10CM:E11 | Type 2 diabetes mellitus |
|  | date constraint | | The terms in this group occurred between Jan 1, 2014 and Jan 1, 2025 | | |
|  | event relationship | | Any instance of Group 2B occurred on or before the first instance of Group 2A | | |
|  | **Group 2B** | | | | |
|  | cannot have |  | demographics | Deceased | Deceased |
|  |  | or | diagnosis | UMLS:ICD10CM:I50 | Heart failure |
|  |  | or | diagnosis | UMLS:ICD10CM:I42 | Cardiomyopathy |
|  |  | or | diagnosis | UMLS:ICD10CM:I21 | Acute myocardial infarction |
|  |  | or | diagnosis | UMLS:ICD10CM:I22 | Subsequent ST elevation (STEMI) and non-ST elevation (NSTEMI) myocardial infarction |
|  |  | or | laboratory | TNX:FINDING:2003 | Left Ventricular Ejection Fraction (LVEF) (%) (at most 50.00 %) |
|  |  | or | diagnosis | UMLS:ICD10CM:I60 | Nontraumatic subarachnoid hemorrhage |
|  |  | or | diagnosis | UMLS:ICD10CM:I61 | Nontraumatic intracerebral hemorrhage |
|  |  | or | diagnosis | UMLS:ICD10CM:I62 | Other and unspecified nontraumatic intracranial hemorrhage |
|  |  | or | diagnosis | UMLS:ICD10CM:I63 | Cerebral infarction |
|  |  | or | diagnosis | UMLS:ICD10CM:G45 | Transient cerebral ischemic attacks and related syndromes |
|  |  | or | diagnosis | UMLS:ICD10CM:I67.82 | Cerebral ischemia |
|  |  | or | diagnosis | UMLS:ICD10CM:I20.0 | Unstable angina |
|  |  | or | procedure | UMLS:CPT:92920 | Percutaneous transluminal coronary angioplasty; single major coronary artery or branch |
|  |  | or | procedure | UMLS:CPT:92928 | Percutaneous transcatheter placement of intracoronary stent(s), with coronary angioplasty when performed; single major coronary artery or branch |
|  |  | or | procedure | UMLS:CPT:92929 | Percutaneous transcatheter placement of intracoronary stent(s), with coronary angioplasty when performed; each additional branch of a major coronary artery (List separately in addition to code for primary procedure) |
|  |  | or | procedure | UMLS:CPT:92921 | Percutaneous transluminal coronary angioplasty; each additional branch of a major coronary artery (List separately in addition to code for primary procedure) |
|  |  | or | procedure | UMLS:CPT:92924 | Percutaneous transluminal coronary atherectomy, with coronary angioplasty when performed; single major coronary artery or branch |
|  |  | or | procedure | UMLS:CPT:92925 | Percutaneous transluminal coronary atherectomy, with coronary angioplasty when performed; each additional branch of a major coronary artery (List separately in addition to code for primary procedure) |
|  |  | or | procedure | UMLS:CPT:92933 | Percutaneous transluminal coronary atherectomy, with intracoronary stent, with coronary angioplasty when performed; single major coronary artery or branch |
|  |  | or | procedure | UMLS:CPT:92934 | Percutaneous transluminal coronary atherectomy, with intracoronary stent, with coronary angioplasty when performed; each additional branch of a major coronary artery (List separately in addition to code for primary procedure) |
|  |  | or | procedure | UMLS:CPT:92937 | Percutaneous transluminal revascularization of or through coronary artery bypass graft (internal mammary, free arterial, venous), any combination of intracoronary stent, atherectomy and angioplasty, including distal protection when performed; single vessel |
|  |  | or | procedure | UMLS:CPT:92938 | Percutaneous transluminal revascularization of or through coronary artery bypass graft (internal mammary, free arterial, venous), any combination of intracoronary stent, atherectomy and angioplasty, including distal protection when performed; each additional branch subtended by the bypass graft (List separately in addition to code for primary procedure) |
|  |  | or | procedure | UMLS:CPT:92941 | Percutaneous transluminal revascularization of acute total/subtotal occlusion during acute myocardial infarction, coronary artery or coronary artery bypass graft, any combination of intracoronary stent, atherectomy and angioplasty, including aspiration thrombectomy when performed, single vessel |
|  |  | or | procedure | UMLS:CPT:92943 | Percutaneous transluminal revascularization of chronic total occlusion, coronary artery, coronary artery branch, or coronary artery bypass graft, any combination of intracoronary stent, atherectomy and angioplasty; single vessel |
|  |  | or | procedure | UMLS:CPT:92944 | Percutaneous transluminal revascularization of chronic total occlusion, coronary artery, coronary artery branch, or coronary artery bypass graft, any combination of intracoronary stent, atherectomy and angioplasty; each additional coronary artery, coronary artery branch, or bypass graft (List separately in addition to code for primary procedure) |
|  |  | or | procedure | UMLS:CPT:92978 | Endoluminal imaging of coronary vessel or graft using intravascular ultrasound (IVUS) or optical coherence tomography (OCT) during diagnostic evaluation and/or therapeutic intervention including imaging supervision, interpretation and report; initial vessel (List separately in addition to code for primary procedure) |
|  |  | or | procedure | UMLS:CPT:92979 | Endoluminal imaging of coronary vessel or graft using intravascular ultrasound (IVUS) or optical coherence tomography (OCT) during diagnostic evaluation and/or therapeutic intervention including imaging supervision, interpretation and report; each additional vessel (List separately in addition to code for primary procedure) |
|  |  | or | procedure | UMLS:CPT:92973 | Percutaneous transluminal coronary thrombectomy mechanical (List separately in addition to code for primary procedure) |
|  |  | or | procedure | UMLS:CPT:92977 | Thrombolysis, coronary; by intravenous infusion |
|  |  | or | procedure | UMLS:CPT:92974 | Transcatheter placement of radiation delivery device for subsequent coronary intravascular brachytherapy (List separately in addition to code for primary procedure) |
|  |  | or | procedure | UMLS:CPT:92975 | Thrombolysis, coronary; by intracoronary infusion, including selective coronary angiography |
|  |  | or | procedure | UMLS:CPT:93571 | Intravascular Doppler velocity and/or pressure derived coronary flow reserve measurement (coronary vessel or graft) during coronary angiography including pharmacologically induced stress; initial vessel (List separately in addition to code for primary procedure) |
|  |  | or | procedure | UMLS:CPT:93572 | Intravascular Doppler velocity and/or pressure derived coronary flow reserve measurement (coronary vessel or graft) during coronary angiography including pharmacologically induced stress; each additional vessel (List separately in addition to code for primary procedure) |
|  |  | or | procedure | UMLS:HCPCS:C9600 | Percutaneous transcatheter placement of drug eluting intracoronary stent(s), with coronary angioplasty when performed; single major coronary artery or branch |
|  |  | or | procedure | UMLS:HCPCS:C9606 | Percutaneous transluminal revascularization of acute total/subtotal occlusion during acute myocardial infarction, coronary artery or coronary artery bypass graft, any combination of drug-eluting intracoronary stent, atherectomy and angioplasty, including aspiration thrombectomy when performed, single vessel |
|  |  | or | procedure | UMLS:HCPCS:C9601 | Percutaneous transcatheter placement of drug-eluting intracoronary stent(s), with coronary angioplasty when performed; each additional branch of a major coronary artery (list separately in addition to code for primary procedure) |
|  |  | or | procedure | UMLS:HCPCS:C9602 | Percutaneous transluminal coronary atherectomy, with drug eluting intracoronary stent, with coronary angioplasty when performed; single major coronary artery or branch |
|  |  | or | procedure | UMLS:HCPCS:C9604 | Percutaneous transluminal revascularization of or through coronary artery bypass graft (internal mammary, free arterial, venous), any combination of drug-eluting intracoronary stent, atherectomy and angioplasty, including distal protection when performed; single vessel |
|  |  | or | procedure | UMLS:HCPCS:C9607 | Percutaneous transluminal revascularization of chronic total occlusion, coronary artery, coronary artery branch, or coronary artery bypass graft, any combination of drug-eluting intracoronary stent, atherectomy and angioplasty; single vessel |
|  |  | or | procedure | UMLS:HCPCS:C9603 | Percutaneous transluminal coronary atherectomy, with drug-eluting intracoronary stent, with coronary angioplasty when performed; each additional branch of a major coronary artery (list separately in addition to code for primary procedure) |
|  |  | or | procedure | UMLS:HCPCS:C9605 | Percutaneous transluminal revascularization of or through coronary artery bypass graft (internal mammary, free arterial, venous), any combination of drug-eluting intracoronary stent, atherectomy and angioplasty, including distal protection when performed; each additional branch subtended by the bypass graft (list separately in addition to code for primary procedure) |
|  |  | or | procedure | UMLS:HCPCS:C9608 | Percutaneous transluminal revascularization of chronic total occlusion, coronary artery, coronary artery branch, or coronary artery bypass graft, any combination of drug-eluting intracoronary stent, atherectomy and angioplasty; each additional coronary artery, coronary artery branch, or bypass graft (list separately in addition to code for primary procedure) |
|  |  | or | diagnosis | UMLS:ICD10CM:N18.6 | End stage renal disease |
|  |  | or | diagnosis | UMLS:ICD10CM:Z99.2 | Dependence on renal dialysis |
|  |  | or | procedure | UMLS:CPT:1012740 | Dialysis Services and Procedures |
|  |  | or | medication | NLM:ATC:A10BJ | Glucagon-like peptide-1 (GLP-1) analogues |
|  |  | or | medication | NLM:RXNORM:2601723 | tirzepatide |
|  |  | or | medication | NLM:ATC:A10BH | Dipeptidyl peptidase 4 (DPP-4) inhibitors |
|  |  | or | medication | NLM:VA:HS501 | INSULIN |
|  |  | or | medication | NLM:ATC:A10BK | Sodium-glucose co-transporter 2 (SGLT2) inhibitors |
|  |  | or | medication | NLM:ATC:A10BG | Thiazolidinediones |
|  |  | or | medication | NLM:ATC:A10BB | Sulfonylureas |
|  |  | or | diagnosis | UMLS:ICD10CM:Z95 | Presence of cardiac and vascular implants and grafts |
|  |  | or | diagnosis | UMLS:ICD10CM:Z98.61 | Coronary angioplasty status |
| Group 3 | | | | | |
|  | **Group 3A** | | | | |
|  | must have | any of | diagnosis | UMLS:ICD10CM:M06 | Other rheumatoid arthritis |
|  |  |  | diagnosis | UMLS:ICD10CM:M05 | Rheumatoid arthritis with rheumatoid factor |
|  |  | and | laboratory | TNX:9083 | BMI (at least 30.00 kg/m2) |
|  |  | and | diagnosis | UMLS:ICD10CM:E11 | Type 2 diabetes mellitus |
|  | date constraint | | The terms in this group occurred between Jan 1, 2014 and Jan 1, 2025 | | |
|  | event relationship | | Any instance of Group 3B occurred within 6 months on or before any instance of Group 3A | | |
|  | **Group 3B** | | | | |
|  | must have | any of | diagnosis | UMLS:ICD10CM:M06 | Other rheumatoid arthritis |
|  |  |  | diagnosis | UMLS:ICD10CM:M05 | Rheumatoid arthritis with rheumatoid factor |

### Query Criteria for Cohort 2 (query name: non-GLP1RA)

This query was run on the network US Collaborative Network with 67 HCO(s) queried and 67 HCO(s) responded. A total of 49 provider(s) responded with patients. The final cohort included 2,972 patients who matched the query criteria listed in the table below.

| Group 1 | | | | | |
| --- | --- | --- | --- | --- | --- |
|  | **Group 1A** | | | | |
|  | must have | any of | diagnosis | UMLS:ICD10CM:M06 | Other rheumatoid arthritis |
|  |  |  | diagnosis | UMLS:ICD10CM:M05 | Rheumatoid arthritis with rheumatoid factor |
|  |  | and | diagnosis | UMLS:ICD10CM:E11 | Type 2 diabetes mellitus |
|  |  | and | laboratory | TNX:9083 | BMI (at least 30.00 kg/m2) |
|  | date constraint | | The terms in this group occurred between Jan 1, 2014 and Jan 1, 2025 | | |
|  | event relationship | | Any instance of Group 1B occurred within 3 months on or after any instance of Group 1A | | |
|  | **Group 1B** | | | | |
|  | must have | any of | medication | NLM:ATC:A10BH | Dipeptidyl peptidase 4 (DPP-4) inhibitors |
|  |  |  | medication | NLM:ATC:A10BK | Sodium-glucose co-transporter 2 (SGLT2) inhibitors |
|  |  |  | medication | NLM:ATC:A10BB | Sulfonylureas |
|  |  |  | medication | NLM:ATC:A10BG | Thiazolidinediones |
|  | cannot have |  | medication | NLM:ATC:A10BJ | Glucagon-like peptide-1 (GLP-1) analogues |
|  |  | or | medication | NLM:RXNORM:2601723 | tirzepatide |
|  |  | or | visit | UMLS:HL7V3.0:VisitType:IMP | Visit: Inpatient Encounter |
|  |  | or | medication | NLM:VA:HS501 | INSULIN |
| Group 2 | | | | | |
|  | **Group 2A** | | | | |
|  | must have | any of | diagnosis | UMLS:ICD10CM:M06 | Other rheumatoid arthritis |
|  |  |  | diagnosis | UMLS:ICD10CM:M05 | Rheumatoid arthritis with rheumatoid factor |
|  |  | and | diagnosis | UMLS:ICD10CM:E11 | Type 2 diabetes mellitus |
|  |  | and | laboratory | TNX:9083 | BMI (at least 30.00 kg/m2) |
|  | date constraint | | The terms in this group occurred between Jan 1, 2014 and Jan 1, 2025 | | |
|  | event relationship | | Any instance of Group 2B occurred on or before the first instance of Group 2A | | |
|  | **Group 2B** | | | | |
|  | cannot have |  | demographics | Deceased | Deceased |
|  |  | or | diagnosis | UMLS:ICD10CM:I50 | Heart failure |
|  |  | or | diagnosis | UMLS:ICD10CM:I42 | Cardiomyopathy |
|  |  | or | diagnosis | UMLS:ICD10CM:I21 | Acute myocardial infarction |
|  |  | or | diagnosis | UMLS:ICD10CM:I22 | Subsequent ST elevation (STEMI) and non-ST elevation (NSTEMI) myocardial infarction |
|  |  | or | laboratory | TNX:FINDING:2003 | Left Ventricular Ejection Fraction (LVEF) (%) (at most 50.00 %) |
|  |  | or | diagnosis | UMLS:ICD10CM:I60 | Nontraumatic subarachnoid hemorrhage |
|  |  | or | diagnosis | UMLS:ICD10CM:I61 | Nontraumatic intracerebral hemorrhage |
|  |  | or | diagnosis | UMLS:ICD10CM:I62 | Other and unspecified nontraumatic intracranial hemorrhage |
|  |  | or | diagnosis | UMLS:ICD10CM:I63 | Cerebral infarction |
|  |  | or | diagnosis | UMLS:ICD10CM:G45 | Transient cerebral ischemic attacks and related syndromes |
|  |  | or | diagnosis | UMLS:ICD10CM:I67.82 | Cerebral ischemia |
|  |  | or | diagnosis | UMLS:ICD10CM:I20.0 | Unstable angina |
|  |  | or | procedure | UMLS:CPT:92920 | Percutaneous transluminal coronary angioplasty; single major coronary artery or branch |
|  |  | or | procedure | UMLS:CPT:92928 | Percutaneous transcatheter placement of intracoronary stent(s), with coronary angioplasty when performed; single major coronary artery or branch |
|  |  | or | procedure | UMLS:CPT:92929 | Percutaneous transcatheter placement of intracoronary stent(s), with coronary angioplasty when performed; each additional branch of a major coronary artery (List separately in addition to code for primary procedure) |
|  |  | or | procedure | UMLS:CPT:92921 | Percutaneous transluminal coronary angioplasty; each additional branch of a major coronary artery (List separately in addition to code for primary procedure) |
|  |  | or | procedure | UMLS:CPT:92924 | Percutaneous transluminal coronary atherectomy, with coronary angioplasty when performed; single major coronary artery or branch |
|  |  | or | procedure | UMLS:CPT:92925 | Percutaneous transluminal coronary atherectomy, with coronary angioplasty when performed; each additional branch of a major coronary artery (List separately in addition to code for primary procedure) |
|  |  | or | procedure | UMLS:CPT:92933 | Percutaneous transluminal coronary atherectomy, with intracoronary stent, with coronary angioplasty when performed; single major coronary artery or branch |
|  |  | or | procedure | UMLS:CPT:92934 | Percutaneous transluminal coronary atherectomy, with intracoronary stent, with coronary angioplasty when performed; each additional branch of a major coronary artery (List separately in addition to code for primary procedure) |
|  |  | or | procedure | UMLS:CPT:92937 | Percutaneous transluminal revascularization of or through coronary artery bypass graft (internal mammary, free arterial, venous), any combination of intracoronary stent, atherectomy and angioplasty, including distal protection when performed; single vessel |
|  |  | or | procedure | UMLS:CPT:92938 | Percutaneous transluminal revascularization of or through coronary artery bypass graft (internal mammary, free arterial, venous), any combination of intracoronary stent, atherectomy and angioplasty, including distal protection when performed; each additional branch subtended by the bypass graft (List separately in addition to code for primary procedure) |
|  |  | or | procedure | UMLS:CPT:92941 | Percutaneous transluminal revascularization of acute total/subtotal occlusion during acute myocardial infarction, coronary artery or coronary artery bypass graft, any combination of intracoronary stent, atherectomy and angioplasty, including aspiration thrombectomy when performed, single vessel |
|  |  | or | procedure | UMLS:CPT:92943 | Percutaneous transluminal revascularization of chronic total occlusion, coronary artery, coronary artery branch, or coronary artery bypass graft, any combination of intracoronary stent, atherectomy and angioplasty; single vessel |
|  |  | or | procedure | UMLS:CPT:92944 | Percutaneous transluminal revascularization of chronic total occlusion, coronary artery, coronary artery branch, or coronary artery bypass graft, any combination of intracoronary stent, atherectomy and angioplasty; each additional coronary artery, coronary artery branch, or bypass graft (List separately in addition to code for primary procedure) |
|  |  | or | procedure | UMLS:CPT:92978 | Endoluminal imaging of coronary vessel or graft using intravascular ultrasound (IVUS) or optical coherence tomography (OCT) during diagnostic evaluation and/or therapeutic intervention including imaging supervision, interpretation and report; initial vessel (List separately in addition to code for primary procedure) |
|  |  | or | procedure | UMLS:CPT:92979 | Endoluminal imaging of coronary vessel or graft using intravascular ultrasound (IVUS) or optical coherence tomography (OCT) during diagnostic evaluation and/or therapeutic intervention including imaging supervision, interpretation and report; each additional vessel (List separately in addition to code for primary procedure) |
|  |  | or | procedure | UMLS:CPT:92973 | Percutaneous transluminal coronary thrombectomy mechanical (List separately in addition to code for primary procedure) |
|  |  | or | procedure | UMLS:CPT:92977 | Thrombolysis, coronary; by intravenous infusion |
|  |  | or | procedure | UMLS:CPT:92974 | Transcatheter placement of radiation delivery device for subsequent coronary intravascular brachytherapy (List separately in addition to code for primary procedure) |
|  |  | or | procedure | UMLS:CPT:92975 | Thrombolysis, coronary; by intracoronary infusion, including selective coronary angiography |
|  |  | or | procedure | UMLS:CPT:93571 | Intravascular Doppler velocity and/or pressure derived coronary flow reserve measurement (coronary vessel or graft) during coronary angiography including pharmacologically induced stress; initial vessel (List separately in addition to code for primary procedure) |
|  |  | or | procedure | UMLS:CPT:93572 | Intravascular Doppler velocity and/or pressure derived coronary flow reserve measurement (coronary vessel or graft) during coronary angiography including pharmacologically induced stress; each additional vessel (List separately in addition to code for primary procedure) |
|  |  | or | procedure | UMLS:HCPCS:C9600 | Percutaneous transcatheter placement of drug eluting intracoronary stent(s), with coronary angioplasty when performed; single major coronary artery or branch |
|  |  | or | procedure | UMLS:HCPCS:C9606 | Percutaneous transluminal revascularization of acute total/subtotal occlusion during acute myocardial infarction, coronary artery or coronary artery bypass graft, any combination of drug-eluting intracoronary stent, atherectomy and angioplasty, including aspiration thrombectomy when performed, single vessel |
|  |  | or | procedure | UMLS:HCPCS:C9601 | Percutaneous transcatheter placement of drug-eluting intracoronary stent(s), with coronary angioplasty when performed; each additional branch of a major coronary artery (list separately in addition to code for primary procedure) |
|  |  | or | procedure | UMLS:HCPCS:C9602 | Percutaneous transluminal coronary atherectomy, with drug eluting intracoronary stent, with coronary angioplasty when performed; single major coronary artery or branch |
|  |  | or | procedure | UMLS:HCPCS:C9604 | Percutaneous transluminal revascularization of or through coronary artery bypass graft (internal mammary, free arterial, venous), any combination of drug-eluting intracoronary stent, atherectomy and angioplasty, including distal protection when performed; single vessel |
|  |  | or | procedure | UMLS:HCPCS:C9607 | Percutaneous transluminal revascularization of chronic total occlusion, coronary artery, coronary artery branch, or coronary artery bypass graft, any combination of drug-eluting intracoronary stent, atherectomy and angioplasty; single vessel |
|  |  | or | procedure | UMLS:HCPCS:C9603 | Percutaneous transluminal coronary atherectomy, with drug-eluting intracoronary stent, with coronary angioplasty when performed; each additional branch of a major coronary artery (list separately in addition to code for primary procedure) |
|  |  | or | procedure | UMLS:HCPCS:C9605 | Percutaneous transluminal revascularization of or through coronary artery bypass graft (internal mammary, free arterial, venous), any combination of drug-eluting intracoronary stent, atherectomy and angioplasty, including distal protection when performed; each additional branch subtended by the bypass graft (list separately in addition to code for primary procedure) |
|  |  | or | procedure | UMLS:HCPCS:C9608 | Percutaneous transluminal revascularization of chronic total occlusion, coronary artery, coronary artery branch, or coronary artery bypass graft, any combination of drug-eluting intracoronary stent, atherectomy and angioplasty; each additional coronary artery, coronary artery branch, or bypass graft (list separately in addition to code for primary procedure) |
|  |  | or | diagnosis | UMLS:ICD10CM:N18.6 | End stage renal disease |
|  |  | or | diagnosis | UMLS:ICD10CM:Z99.2 | Dependence on renal dialysis |
|  |  | or | procedure | UMLS:CPT:1012740 | Dialysis Services and Procedures |
|  |  | or | medication | NLM:ATC:A10BJ | Glucagon-like peptide-1 (GLP-1) analogues |
|  |  | or | medication | NLM:RXNORM:2601723 | tirzepatide |
|  |  | or | medication | NLM:ATC:A10BH | Dipeptidyl peptidase 4 (DPP-4) inhibitors |
|  |  | or | medication | NLM:ATC:A10BB | Sulfonylureas |
|  |  | or | medication | NLM:ATC:A10BK | Sodium-glucose co-transporter 2 (SGLT2) inhibitors |
|  |  | or | medication | NLM:ATC:A10BG | Thiazolidinediones |
|  |  | or | medication | NLM:VA:HS501 | INSULIN |
|  |  | or | diagnosis | UMLS:ICD10CM:Z95 | Presence of cardiac and vascular implants and grafts |
|  |  | or | diagnosis | UMLS:ICD10CM:Z98.61 | Coronary angioplasty status |

**eMethods 3. Outcome Definitions**

Table below outlines the definitions for each outcome and the analysis specifications. For outcome definitions consisting of more than one term, at least one term must match.

| MACCE | | | | | | | | | | |
| --- | --- | --- | --- | --- | --- | --- | --- | --- | --- | --- |
|  | **Outcome definition** | | | | | | | | | |
|  | | Demographics | | | Deceased | | | | | Deceased |
|  | | Diagnosis | | | UMLS:ICD10CM:I21 | | | | | Acute myocardial infarction |
|  | | Diagnosis | | | UMLS:ICD10CM:I22 | | | | | Subsequent ST elevation (STEMI) and non-ST elevation (NSTEMI) myocardial infarction |
|  | | Diagnosis | | | UMLS:ICD10CM:I63 | | | | | Cerebral infarction |
|  | | Diagnosis | | | UMLS:ICD10CM:I67.82 | | | | | Cerebral ischemia |
|  | | Diagnosis | | | UMLS:ICD10CM:I60 | | | | | Nontraumatic subarachnoid hemorrhage |
|  | | Diagnosis | | | UMLS:ICD10CM:I61 | | | | | Nontraumatic intracerebral hemorrhage |
|  | | Diagnosis | | | UMLS:ICD10CM:I62 | | | | | Other and unspecified nontraumatic intracranial hemorrhage |
|  | | Diagnosis | | | UMLS:ICD10CM:I50 | | | | | Heart failure |
|  | | Diagnosis | | | UMLS:ICD10CM:I42 | | | | | Cardiomyopathy |
|  | | Laboratory | | | TNX:FINDING:2003 | | | | | Left Ventricular Ejection Fraction (LVEF) (%) (at most 50.00 % (most recent occurrence)) |
|  | | Diagnosis | | | UMLS:ICD10CM:G45 | | | | | Transient cerebral ischemic attacks and related syndromes |
|  | | Diagnosis | | | UMLS:ICD10CM:I43 | | | | | Cardiomyopathy in diseases classified elsewhere |
|  | | Medication | | | NLM:ATC:C03 | | | | | DIURETICS (Route: Injectable Product) |
|  | | Medication | | | NLM:RXNORM:1656328 | | | | | sacubitril |
|  | | Diagnosis | | | UMLS:ICD10CM:I20.0 | | | | | Unstable angina |
| Death | | | | | | | | | | |
|  | **Outcome definition** | | | | | | | | | |
|  | | Demographics | | | Deceased | | | | | Deceased |
| New HF | | | | | | | | | | |
|  | **Outcome definition** | | | | | | | | | |
|  | | Diagnosis | | | UMLS:ICD10CM:I50 | | | | | Heart failure |
|  | | Diagnosis | | | UMLS:ICD10CM:I42 | | | | | Cardiomyopathy |
|  | | Laboratory | | | TNX:FINDING:2003 | | | | | Left Ventricular Ejection Fraction (LVEF) (%) (at most 50.00 % (most recent occurrence)) |
|  | | Diagnosis | | | UMLS:ICD10CM:I43 | | | | | Cardiomyopathy in diseases classified elsewhere |
|  | | Medication | | | NLM:ATC:C03 | | | | | DIURETICS (Route: Injectable Product) |
|  | | Medication | | | NLM:RXNORM:1656328 | | | | | sacubitril |
| HF hospitalization | | | | | | | | | | |
|  | **Outcome definition** | | | | | | | | | |
|  | | Diagnosis | | | UMLS:ICD10CM:I50.33 | | | | | Acute on chronic diastolic (congestive) heart failure |
|  | | Diagnosis | | | UMLS:ICD10CM:I50.23 | | | | | Acute on chronic systolic (congestive) heart failure |
|  | | Diagnosis | | | UMLS:ICD10CM:I50.21 | | | | | Acute systolic (congestive) heart failure |
|  | | Diagnosis | | | UMLS:ICD10CM:I50.43 | | | | | Acute on chronic combined systolic (congestive) and diastolic (congestive) heart failure |
|  | | Diagnosis | | | UMLS:ICD10CM:I50.31 | | | | | Acute diastolic (congestive) heart failure |
|  | | Diagnosis | | | UMLS:ICD10CM:I50.41 | | | | | Acute combined systolic (congestive) and diastolic (congestive) heart failure |
|  | | Medication | | | NLM:ATC:C03 | | | | | DIURETICS (Route: Injectable Product) |
| AMI | | | | | | | | | | |
|  | **Outcome definition** | | | | | | | | | |
|  | | Diagnosis | | | UMLS:ICD10CM:I21 | | | | | Acute myocardial infarction |
|  | | Diagnosis | | | UMLS:ICD10CM:I22 | | | | | Subsequent ST elevation (STEMI) and non-ST elevation (NSTEMI) myocardial infarction |
| Any stroke | | | | | | | | | | |
|  | **Outcome definition** | | | | | | | | | |
|  | | Diagnosis | | | UMLS:ICD10CM:I63 | | | | | Cerebral infarction |
|  | | Diagnosis | | | UMLS:ICD10CM:G45 | | | | | Transient cerebral ischemic attacks and related syndromes |
|  | | Diagnosis | | | UMLS:ICD10CM:I67.82 | | | | | Cerebral ischemia |
|  | | Diagnosis | | | UMLS:ICD10CM:I60 | | | | | Nontraumatic subarachnoid hemorrhage |
|  | | Diagnosis | | | UMLS:ICD10CM:I61 | | | | | Nontraumatic intracerebral hemorrhage |
|  | | Diagnosis | | | UMLS:ICD10CM:I62 | | | | | Other and unspecified nontraumatic intracranial hemorrhage |
| Second-line DMARDs | | | | | | | | | | |
|  | **Outcome definition** | | | | | | | | | |
|  | | | | Medication | | | NLM:RXNORM:612865 | tocilizumab | | |
|  | | | | Medication | | | NLM:RXNORM:614391 | abatacept | | |
|  | | | | Medication | | | NLM:RXNORM:121191 | rituximab | | |
|  | | | | Medication | | | NLM:RXNORM:72435 | anakinra | | |
|  | | | | Medication | | | NLM:ATC:L04AB | Tumor necrosis factor alpha (TNF-alpha) inhibitors | | |
|  | | | | Medication | | | NLM:ATC:L04AF | Janus-associated kinase (JAK) inhibitors | | |
| Falsification 1: UTI | | | | | | | | | | |
|  | **Outcome definition** | | | | | | | | | |
|  | | | Diagnosis | | | UMLS:ICD10CM:N39.0 | | | Urinary tract infection, site not specified | |
| Falsification 2: fracture | | | | | | | | | | |
|  | **Outcome definition** | | | | | | | | | |
|  | | | Diagnosis | | | UMLS:ICD10CM:S02 | | | Fracture of skull and facial bones | |
|  | | | Diagnosis | | | UMLS:ICD10CM:S22 | | | Fracture of rib(s), sternum and thoracic spine | |
|  | | | Diagnosis | | | UMLS:ICD10CM:S42 | | | Fracture of shoulder and upper arm | |
|  | | | Diagnosis | | | UMLS:ICD10CM:S52 | | | Fracture of forearm | |
|  | | | Diagnosis | | | UMLS:ICD10CM:S72 | | | Fracture of femur | |
|  | | | Diagnosis | | | UMLS:ICD10CM:S82 | | | Fracture of lower leg, including ankle | |
|  | | | Diagnosis | | | UMLS:ICD10CM:S62 | | | Fracture at wrist and hand level | |
|  | | | Diagnosis | | | UMLS:ICD10CM:S92 | | | Fracture of foot and toe, except ankle | |
|  | | | Diagnosis | | | UMLS:ICD10CM:S12 | | | Fracture of cervical vertebra and other parts of neck | |
|  | | | Diagnosis | | | UMLS:ICD10CM:S32 | | | Fracture of lumbar spine and pelvis | |
|  | | | Diagnosis | | | UMLS:ICD10CM:S62 | | | Fracture at wrist and hand level | |

**eMethods 4. Non-RA Cohort Query**

This query was run on the network US Collaborative Network with 67 HCO(s) queried and 67 HCO(s) responded. A total of 60 provider(s) responded with patients. The final cohort included 127,106 patients who matched the query criteria listed in the table below.

| Group 1 | | | | | |
| --- | --- | --- | --- | --- | --- |
|  | **Group 1A** | | | | |
|  | must have |  | diagnosis | UMLS:ICD10CM:E11 | Type 2 diabetes mellitus |
|  |  | and | laboratory | TNX:9083 | BMI (at least 30.00 kg/m2) |
|  | cannot have |  | diagnosis | UMLS:ICD10CM:M06 | Other rheumatoid arthritis |
|  |  | or | diagnosis | UMLS:ICD10CM:M05 | Rheumatoid arthritis with rheumatoid factor |
|  | date constraint | | The terms in this group occurred between Jan 1, 2014 and Jan 1, 2025 | | |
|  | event relationship | | Any instance of Group 1B occurred within 3 months on or after any instance of Group 1A | | |
|  | **Group 1B** | | | | |
|  | must have | any of | medication | NLM:ATC:A10BH | Dipeptidyl peptidase 4 (DPP-4) inhibitors |
|  |  |  | medication | NLM:ATC:A10BK | Sodium-glucose co-transporter 2 (SGLT2) inhibitors |
|  |  |  | medication | NLM:ATC:A10BB | Sulfonylureas |
|  |  |  | medication | NLM:ATC:A10BG | Thiazolidinediones |
|  | cannot have |  | medication | NLM:ATC:A10BJ | Glucagon-like peptide-1 (GLP-1) analogues |
|  |  | or | medication | NLM:RXNORM:2601723 | tirzepatide |
|  |  | or | visit | UMLS:HL7V3.0:VisitType:IMP | Visit: Inpatient Encounter |
|  |  | or | medication | NLM:VA:HS501 | INSULIN |
| Group 2 | | | | | |
|  | **Group 2A** | | | | |
|  | must have |  | diagnosis | UMLS:ICD10CM:E11 | Type 2 diabetes mellitus |
|  |  | and | laboratory | TNX:9083 | BMI (at least 30.00 kg/m2) |
|  | cannot have |  | diagnosis | UMLS:ICD10CM:M06 | Other rheumatoid arthritis |
|  |  | or | diagnosis | UMLS:ICD10CM:M05 | Rheumatoid arthritis with rheumatoid factor |
|  | date constraint | | The terms in this group occurred between Jan 1, 2014 and Jan 1, 2025 | | |
|  | event relationship | | Any instance of Group 2B occurred on or before the first instance of Group 2A | | |
|  | **Group 2B** | | | | |
|  | cannot have |  | demographics | Deceased | Deceased |
|  |  | or | diagnosis | UMLS:ICD10CM:I50 | Heart failure |
|  |  | or | diagnosis | UMLS:ICD10CM:I42 | Cardiomyopathy |
|  |  | or | diagnosis | UMLS:ICD10CM:I21 | Acute myocardial infarction |
|  |  | or | diagnosis | UMLS:ICD10CM:I22 | Subsequent ST elevation (STEMI) and non-ST elevation (NSTEMI) myocardial infarction |
|  |  | or | laboratory | TNX:FINDING:2003 | Left Ventricular Ejection Fraction (LVEF) (%) (at most 50.00 %) |
|  |  | or | diagnosis | UMLS:ICD10CM:I60 | Nontraumatic subarachnoid hemorrhage |
|  |  | or | diagnosis | UMLS:ICD10CM:I61 | Nontraumatic intracerebral hemorrhage |
|  |  | or | diagnosis | UMLS:ICD10CM:I62 | Other and unspecified nontraumatic intracranial hemorrhage |
|  |  | or | diagnosis | UMLS:ICD10CM:I63 | Cerebral infarction |
|  |  | or | diagnosis | UMLS:ICD10CM:G45 | Transient cerebral ischemic attacks and related syndromes |
|  |  | or | diagnosis | UMLS:ICD10CM:I67.82 | Cerebral ischemia |
|  |  | or | diagnosis | UMLS:ICD10CM:I20.0 | Unstable angina |
|  |  | or | procedure | UMLS:CPT:92920 | Percutaneous transluminal coronary angioplasty; single major coronary artery or branch |
|  |  | or | procedure | UMLS:CPT:92928 | Percutaneous transcatheter placement of intracoronary stent(s), with coronary angioplasty when performed; single major coronary artery or branch |
|  |  | or | procedure | UMLS:CPT:92929 | Percutaneous transcatheter placement of intracoronary stent(s), with coronary angioplasty when performed; each additional branch of a major coronary artery (List separately in addition to code for primary procedure) |
|  |  | or | procedure | UMLS:CPT:92921 | Percutaneous transluminal coronary angioplasty; each additional branch of a major coronary artery (List separately in addition to code for primary procedure) |
|  |  | or | procedure | UMLS:CPT:92924 | Percutaneous transluminal coronary atherectomy, with coronary angioplasty when performed; single major coronary artery or branch |
|  |  | or | procedure | UMLS:CPT:92925 | Percutaneous transluminal coronary atherectomy, with coronary angioplasty when performed; each additional branch of a major coronary artery (List separately in addition to code for primary procedure) |
|  |  | or | procedure | UMLS:CPT:92933 | Percutaneous transluminal coronary atherectomy, with intracoronary stent, with coronary angioplasty when performed; single major coronary artery or branch |
|  |  | or | procedure | UMLS:CPT:92934 | Percutaneous transluminal coronary atherectomy, with intracoronary stent, with coronary angioplasty when performed; each additional branch of a major coronary artery (List separately in addition to code for primary procedure) |
|  |  | or | procedure | UMLS:CPT:92937 | Percutaneous transluminal revascularization of or through coronary artery bypass graft (internal mammary, free arterial, venous), any combination of intracoronary stent, atherectomy and angioplasty, including distal protection when performed; single vessel |
|  |  | or | procedure | UMLS:CPT:92938 | Percutaneous transluminal revascularization of or through coronary artery bypass graft (internal mammary, free arterial, venous), any combination of intracoronary stent, atherectomy and angioplasty, including distal protection when performed; each additional branch subtended by the bypass graft (List separately in addition to code for primary procedure) |
|  |  | or | procedure | UMLS:CPT:92941 | Percutaneous transluminal revascularization of acute total/subtotal occlusion during acute myocardial infarction, coronary artery or coronary artery bypass graft, any combination of intracoronary stent, atherectomy and angioplasty, including aspiration thrombectomy when performed, single vessel |
|  |  | or | procedure | UMLS:CPT:92943 | Percutaneous transluminal revascularization of chronic total occlusion, coronary artery, coronary artery branch, or coronary artery bypass graft, any combination of intracoronary stent, atherectomy and angioplasty; single vessel |
|  |  | or | procedure | UMLS:CPT:92944 | Percutaneous transluminal revascularization of chronic total occlusion, coronary artery, coronary artery branch, or coronary artery bypass graft, any combination of intracoronary stent, atherectomy and angioplasty; each additional coronary artery, coronary artery branch, or bypass graft (List separately in addition to code for primary procedure) |
|  |  | or | procedure | UMLS:CPT:92978 | Endoluminal imaging of coronary vessel or graft using intravascular ultrasound (IVUS) or optical coherence tomography (OCT) during diagnostic evaluation and/or therapeutic intervention including imaging supervision, interpretation and report; initial vessel (List separately in addition to code for primary procedure) |
|  |  | or | procedure | UMLS:CPT:92979 | Endoluminal imaging of coronary vessel or graft using intravascular ultrasound (IVUS) or optical coherence tomography (OCT) during diagnostic evaluation and/or therapeutic intervention including imaging supervision, interpretation and report; each additional vessel (List separately in addition to code for primary procedure) |
|  |  | or | procedure | UMLS:CPT:92973 | Percutaneous transluminal coronary thrombectomy mechanical (List separately in addition to code for primary procedure) |
|  |  | or | procedure | UMLS:CPT:92977 | Thrombolysis, coronary; by intravenous infusion |
|  |  | or | procedure | UMLS:CPT:92974 | Transcatheter placement of radiation delivery device for subsequent coronary intravascular brachytherapy (List separately in addition to code for primary procedure) |
|  |  | or | procedure | UMLS:CPT:92975 | Thrombolysis, coronary; by intracoronary infusion, including selective coronary angiography |
|  |  | or | procedure | UMLS:CPT:93571 | Intravascular Doppler velocity and/or pressure derived coronary flow reserve measurement (coronary vessel or graft) during coronary angiography including pharmacologically induced stress; initial vessel (List separately in addition to code for primary procedure) |
|  |  | or | procedure | UMLS:CPT:93572 | Intravascular Doppler velocity and/or pressure derived coronary flow reserve measurement (coronary vessel or graft) during coronary angiography including pharmacologically induced stress; each additional vessel (List separately in addition to code for primary procedure) |
|  |  | or | procedure | UMLS:HCPCS:C9600 | Percutaneous transcatheter placement of drug eluting intracoronary stent(s), with coronary angioplasty when performed; single major coronary artery or branch |
|  |  | or | procedure | UMLS:HCPCS:C9606 | Percutaneous transluminal revascularization of acute total/subtotal occlusion during acute myocardial infarction, coronary artery or coronary artery bypass graft, any combination of drug-eluting intracoronary stent, atherectomy and angioplasty, including aspiration thrombectomy when performed, single vessel |
|  |  | or | procedure | UMLS:HCPCS:C9601 | Percutaneous transcatheter placement of drug-eluting intracoronary stent(s), with coronary angioplasty when performed; each additional branch of a major coronary artery (list separately in addition to code for primary procedure) |
|  |  | or | procedure | UMLS:HCPCS:C9602 | Percutaneous transluminal coronary atherectomy, with drug eluting intracoronary stent, with coronary angioplasty when performed; single major coronary artery or branch |
|  |  | or | procedure | UMLS:HCPCS:C9604 | Percutaneous transluminal revascularization of or through coronary artery bypass graft (internal mammary, free arterial, venous), any combination of drug-eluting intracoronary stent, atherectomy and angioplasty, including distal protection when performed; single vessel |
|  |  | or | procedure | UMLS:HCPCS:C9607 | Percutaneous transluminal revascularization of chronic total occlusion, coronary artery, coronary artery branch, or coronary artery bypass graft, any combination of drug-eluting intracoronary stent, atherectomy and angioplasty; single vessel |
|  |  | or | procedure | UMLS:HCPCS:C9603 | Percutaneous transluminal coronary atherectomy, with drug-eluting intracoronary stent, with coronary angioplasty when performed; each additional branch of a major coronary artery (list separately in addition to code for primary procedure) |
|  |  | or | procedure | UMLS:HCPCS:C9605 | Percutaneous transluminal revascularization of or through coronary artery bypass graft (internal mammary, free arterial, venous), any combination of drug-eluting intracoronary stent, atherectomy and angioplasty, including distal protection when performed; each additional branch subtended by the bypass graft (list separately in addition to code for primary procedure) |
|  |  | or | procedure | UMLS:HCPCS:C9608 | Percutaneous transluminal revascularization of chronic total occlusion, coronary artery, coronary artery branch, or coronary artery bypass graft, any combination of drug-eluting intracoronary stent, atherectomy and angioplasty; each additional coronary artery, coronary artery branch, or bypass graft (list separately in addition to code for primary procedure) |
|  |  | or | diagnosis | UMLS:ICD10CM:N18.6 | End stage renal disease |
|  |  | or | diagnosis | UMLS:ICD10CM:Z99.2 | Dependence on renal dialysis |
|  |  | or | procedure | UMLS:CPT:1012740 | Dialysis Services and Procedures |
|  |  | or | medication | NLM:ATC:A10BJ | Glucagon-like peptide-1 (GLP-1) analogues |
|  |  | or | medication | NLM:RXNORM:2601723 | tirzepatide |
|  |  | or | medication | NLM:ATC:A10BH | Dipeptidyl peptidase 4 (DPP-4) inhibitors |
|  |  | or | medication | NLM:ATC:A10BB | Sulfonylureas |
|  |  | or | medication | NLM:ATC:A10BK | Sodium-glucose co-transporter 2 (SGLT2) inhibitors |
|  |  | or | medication | NLM:ATC:A10BG | Thiazolidinediones |
|  |  | or | medication | NLM:VA:HS501 | INSULIN |
|  |  | or | diagnosis | UMLS:ICD10CM:Z95 | Presence of cardiac and vascular implants and grafts |
|  |  | or | diagnosis | UMLS:ICD10CM:Z98.61 | Coronary angioplasty status |

**eResults 1.** Propensity-score matching

| **Cohort 1 and cohort 2 patient count before and after propensity score matching** | | | | | |
| --- | --- | --- | --- | --- | --- |
|  | | Cohort | Patient count before matching | | Patient count after matching |
|  | | 1 - Semaglutide | 1,200 | | 1,017 |
|  | | 2 - Unnamed | 2,972 | | 1,017 |
| **Propensity score density function - Before and after matching (cohort 1 - purple, cohort 2 - green)** | | | | | |
|  |  |  | |  | |

| **eTable 1: Specification of the Target Trial Protocol and its Emulation** | | |
| --- | --- | --- |
| **Protocol Component** | **Target Trial Specification** | **Observational Study Emulation** |
| **Research Question** | To assess the causal effect of initiating Semaglutide compared to other non-GLP-1 RA glucose-lowering therapy for the primary prevention of major adverse cardiac and cerebrovascular events (MACCE) in adult obese patients with rheumatoid arthritis (RA) and type 2 diabetes mellitus (T2DM) | To emulate the target trial using real-world data from the TriNetX network to estimate the association between Semgalutide initiation and MACCE in obese RA patients with T2DM |
| **Eligibility Criteria** | - Adults (≥18 years) - Documented RA - Documented obesity (BMI ≥30 kg/m^2^). - Documented T2DM (A1c ≥6.5%) - No prior history of MACCE, including stroke, heart failure (HF), acute coronary syndrome (ACS), or coronary revascularization - No prior use of second-line glucose-lowering therapy, including GLP-1 RA, insulin, sodium-glucose co-transporter 2 inhibitors (SGLT2i), dipeptidyl peptidase 4 inhibitors (DPP4i), sulfonylureas, thiazolidinediones (TZD) - No contraindications to GLP-1 RA use: end-stage renal disease (ESRD) or dialysis dependence | - Patients in TriNetX database between Jan 1, 2014 to Oct 1, 2024. - ICD-CM-10 code for RA (M05 or M06) and T2DM (E11) - Documented BMI ≥30 kg/m^2^. - No record of GLP-1 RA, insulin, sulfonylureas, TZD, SGLT2i, and DPP4i prescription prior to Time Zero within TriNetX. - No ICD-10 codes or procedure codes for the following: stroke (I60–63, I67.82, or G45), myocardial infarction (MI) (I21 or I22), unstable angina (I20.0), HF (I42 or I 50), coronary revascularization (Z95, or Z98.61), ESRD (N18.6), or dialysis (Z99.2) prior to Time Zero within TriNetX. |
| **Treatment Strategies** | 1. **Intervention:** Initiation of Semaglutide  2. **Comparator:** Other non-GLP-1 RA second-line glucose-lowering therapy, including SGLT2i, DPP4i, sulfonylureas, or TZD | 1. **Intervention Group:**  Patients with a first-ever prescription record for Semaglutide  2. **Comparator Group:** Patients with a new use of SGLT2i, DPP4i, sulfonylureas, or TZD |
| **Treatment Assignment** | Random assignment at baseline (Time Zero) in a 1:1 ratio to either the Semaglutide or other non-GLP-1 RA second-line glucose-lowering therapies. Open-label. | Emulated by comparing patients who initiated Semaglutide with those who had other non-GLP-1 RA second-line glucose-lowering therapies at Time Zero, after propensity score matching to balance baseline covariates. New-user design. |
| **Start and End of Follow-up (Time Zero)** | **Time Zero:** Date of random assignment. **Start of Follow-up:** Time Zero. **End of Follow-up:** First occurrence of the primary outcome, death, loss to follow-up, or 2 years after Time Zero, whichever occurred first. | **Time Zero (Intervention):** Date of first Semaglutide prescription after meeting all eligibility criteria. **Time Zero (Comparator):** Date of other non-GLP-1 RA second-line glucose-lowering therapies after meeting all eligibility criteria . **Start of Follow-up:** Respective Time Zero for each patient. **End of Follow-up:** Occurrence of an outcome, death, end of data availability in TriNetX for the patient (censoring), or 2 years after Time Zero, whichever occurred first. |
| **Outcomes** | **Primary:** MACCE **Secondary:** All-cause mortality, incident HF, HF hospitalization, MI, and stroke. Outcomes ascertained through systematic follow-up and adjudication if necessary. | **Primary & Secondary:** Defined using ICD-10 codes for diagnoses and relevant procedure codes from EHR data within TriNetX. Mortality data from EHR and linked sources.  **Falsification endpoints:** respiratory and urinary tract infection. |
| **Causal Contrasts of Interest** | Intention-to-treat (ITT) effect: Effect of being assigned to initiate Semaglutide versus assigned to no GLP-1 RA at baseline. | Primarily emulating an ITT-like effect in the matched cohort by comparing outcomes based on initial strategy at Time Zero, regardless of subsequent adherence or crossover (though adherence is not explicitly modeled here beyond initial prescription). |
| **Statistical Analysis Plan (Target Trial)** | - Kaplan-Meier curves for time-to-event outcomes. - Cox proportional hazards regression to estimate Hazard Ratios (HRs) and 95% CIs for the ITT effect. | - Propensity score matching (1:1 nearest neighbor, caliper 0.1 SD) to balance baseline covariates.  - Comparison of baseline characteristics using SMDs (target <0.10). - Cox proportional hazards models in the matched cohort to estimate HRs and 95% CIs. - Bonferroni correction for multiple secondary outcomes.  - E-value calculation for robustness to unmeasured confounding. |
| **Data Source for Emulation** | Not Applicable. | TriNetX Global Collaborative Network (de-identified EHR data from 70 US-based healthcare organizations. |

**eFigure 1.** Illustration of target trial design

| **eTable 2. Cox proportional hazard analysis for Semaglutide use and study endpoints in the PP analysis** | | | | | |
| --- | --- | --- | --- | --- | --- |
|  | Semaglutide  (n = 691) | Non-GLP-1 RA  glucose-lowering agents  (n = 691) | HR  (95% CI) | P-value  (log-rank) | E-value  (LNL) |
| *Primary endpoint* |  |  |  |  |  |
| MACCE, n (%) | 83 (12.0) | 116 (16.9) | 0.68 (0.51 to 0.91) | 0.008 | 2.32 (3.33) |
| *Secondary endpoints* |  |  |  |  |  |
| All-cause mortality, n (%) | <10 | <10 | N/A | N/A |  |
| Incident HF, n (%) | 61 (8.8) | 84 (12.1) | 0.70 (0.50 to 0.97) | 0.03 | 2.21 (3.41) |
| HF hospitalization, n (%) | 11 (1.6) | 25 (3.6) | 0.44 (0.21 to 0.88) | 0.01 | 3.96 (8.99) |
| Myocardial infarction, n (%) | <10 | <10 | N/A | N/A |  |
| Any stroke, n (%) | 15 (2.1) | 23 (3.3) | 0.64 (0.34 to 1.24) | 0.47 |  |
| *Falsification endpoints* |  |  |  |  |  |
| Respiratory infection, n (%) | 173 (25.0) | 149 (21.5) | 1.17 (0.94 to 1.46) | 0.15 |  |
| Fracture, n (%) | 36 (5.2) | 31 (4.5) | 1.19 (0.73 to 1.92) | 0.47 |  |
| *Note:*  *LNL, lower normal limit* | | | | | |

**eFigure 2.** Kaplan-Meier Survival curves for comparative risk of major adverse cardiovascular events between RA and non-RA patients with diabetes who received GLP-1 receptor agonists.

| **eTable 3. Cox proportional hazard analysis comparing RA vs non-RA** | | | | |
| --- | --- | --- | --- | --- |
|  | Patients with RA  (n = 1,198) | Patients without RA  (n = 1,198) | HR  (95% CI) | P-value  (log-rank) |
| *Primary endpoint* |  |  |  |  |
| MACCE, n (%) | 83 (12.0) | 116 (16.9) | 0.68 (0.51 to 0.91) | 0.008 |
| *Secondary endpoints* |  |  |  |  |
| All-cause mortality, n (%) | 12 (1.0) | 20 (1.6) | 0.59 (0.29 to 1.21) | 0.15 |
| Incident HF, n (%) | 111 (9.3) | 151 (12.6) | 0.70 (0.55 to 0.89) | 0.003 |
| HF hospitalization, n (%) | 29 (2.4) | 45 (3.7) | 0.63 (0.39 to 1.00) | 0.05 |
| Myocardial infarction, n (%) | 14 (1.2) | 18 (1.5) | 0.78 (0.39 to 1.57) | 0.48 |
| Any stroke, n (%) | 31 (2.6) | 34 (2.8) | 0.89 (0.55 to 1.45) | 0.65 |
